# TIGIT blockade enhances tumor response to radiotherapy via a CD103 + dendritic cell-dependent mechanism

**DOI:** 10.1007/s00262-022-03227-z

**Published:** 2022-07-06

**Authors:** Kaikai Zhao, Liyang Jiang, Youjiao Si, Shujie Zhou, Zhaoqin Huang, Xiangjiao Meng

**Affiliations:** 1 Department of Radiation Oncology, Shandong Cancer Hospital and Institute, Shandong First Medical University and Shandong Academy of Medical Sciences, Jinan, China; 2 Department of Radiology, Shandong Cancer Hospital and Institute, Shandong First Medical University and Shandong Academy of Medical Sciences, Jinan, China; 3 Cheeloo College of Medicine, Shandong University, Jinan, China; 4 Department of Radiology, Shandong Provincial Hospital Affiliated to Shandong First Medical University, Jinan, China; 5 Department of Radiation Oncology, Yantai Affiliated Hospital of Binzhou Medical University, Yantai, China; 6 Department of Radiology, Yantai Affiliated Hospital of Binzhou Medical University, Yantai, China

**Keywords:** Radiotherapy, TIGIT, Dendritic cell, Immunotherapy, Checkpoint inhibitor

## Abstract

Blockade of the T cell immunoreceptor with the immunoglobulin and immunoreceptor tyrosine-based inhibitory motif domain (TIGIT) can enhance innate and adaptive tumor immunity and radiotherapy (RT) can enhance anti-tumor immunity. However, our data suggest that TIGIT-mediated immune suppression may be an impediment to such goals. Herein, we report on the synergistic effects of RT combined with anti-TIGIT therapy and the mechanism of their interaction. Treatment efficacy was assessed by measuring primary and secondary tumor growth, survival, and immune memory capacity. The function of CD103 + dendritic cells (DCs) under the combined treatment was assessed in wild-type and BATF3-deficient (BATF3^−/−^) mice. FMS-like tyrosine kinase 3 ligand (Flt3L) was used to confirm the role of CD103 + DCs in RT combined with anti-TIGIT therapy. TIGIT was upregulated in immune cells following RT in both esophageal squamous cell carcinoma patients and mouse models. Administration of the anti-TIGIT antibody enhanced the efficacy of RT through a CD8 + T cell-dependent mechanism. It was observed that RT and the anti-TIGIT antibody synergistically enhanced the accumulation of tumor-infiltrating DCs, which activated CD8 + T cells. The efficacy of the combination therapy was negated in the BATF3^−/−^ mouse model. CD103 + DCs were required to promote the anti-tumor effects of combination therapy. Additionally, Flt3L therapy enhanced tumor response to RT combined with TIGIT blockade. Our study demonstrated TIGIT blockade can synergistically enhance anti-tumor T cell responses to RT via CD8 + T cells (dependent on CD103 + DCs), suggesting the clinical potential of targeting the TIGIT pathway and expanding CD103 + DCs in RT.

**Supplementary Information:**

The online version contains supplementary material available at 10.1007/s00262-022-03227-z.

## Introduction

The field of immunotherapy, which has undergone rapid development in the past decade representation an important modality for controlling the progression of malignant tumors [1]. Immune checkpoint inhibitors (ICIs) are promising immunotherapies [2]. However, despite the efficacy of ICIs, many patients fail to respond to treatment, and the majority of patients acquire resistance even after demonstrating good initial responses to immunotherapy [3]. Therefore, intensive research aimed at finding new immune checkpoint targets is ongoing.

TIGIT (T cell immunoreceptor with Ig and ITIM [immunoreceptor tyrosine-based inhibitory motif] domain) was first identified as an immune checkpoint molecule in T cell and natural killer (NK) cell activation in 2009 [4], and was subsequently found to interact with CD155 and CD112 molecules [5]. TIGIT can bridge innate and adaptive immunity and modulate immune responses in autoimmunity, malignancies, and infections [6]. Zhang et al. showed that TIGIT blockade can prevent NK cell exhaustion and elicit potent anti-tumor immunity [7]. TIGIT on T cells can suppress the costimulatory abilities of dendritic cells (DCs) and increase anti-inflammatory cytokines such as IL-10, thus inhibiting T cell responses indirectly by reducing cancer antigen presentation [8]. Additionally, past research has found that TIGIT can inhibit T cell proliferation and function [9–11].

Unfortunately, anti-TIGIT therapy alone has been found to be insufficient for tumor control, researchers speculate that combination therapy could achieve better therapeutic outcomes in a mouse model [12]. The phase II CITYSCAPE trial presented substantial response rates to the TIGIT inhibitor tiragolumab (MTIG7192A, RG-6058) plus atezolizumab only in patients with non-small cell lung cancer as well as programmed death-ligand 1 (PD-L1) tumor proportion scores of 50% or greater [13], suggesting that blockade of the TIGIT checkpoint receptor combined with other treatments deserves further study.

Radiotherapy (RT) can induce tumor cell death and enhance anti-tumor immunity through multiple mechanisms [14]. For example, RT can amplify the cGAS-STING pathway as well as release of the nuclear protein high-mobility group box-1 or can induce expression of chemokines by cancer cells and/or infiltrating immune cells [14]. Combining RT with immunotherapy provides an opportunity to boost systemic tumor response rates, which extends the use of RT to the treatment of both local and extensive disease [15]. However, the underlying mechanisms of RT combined with anti-TIGIT and its optimal combination mode remain unknown [16].

In the current study, we found that TIGIT/CD155 expression was elevated in T cells and DCs after RT. We conducted this study in order to investigate whether blocking the TIGIT/CD155 pathway can enhance the response to RT and/or TIGIT blockade. We demonstrated the following results in the present study. Combination therapy with RT and TIGIT blockade optimized anti-tumor immunity and depletion of CD103 + DCs resulted in a marked reduction in survival and cure rates following combination therapy, suggesting that CD103 + DCs play an important role in regulating treatment response. Therefore, this study provides insights into the mechanism by which RT and anti-TIGIT therapy interact to improve outcomes in cancer patients.

## Materials and methods

### Patient treatment and samples

Twenty-three patients with esophageal squamous cell carcinoma (ESCC) who received neoadjuvant chemoradiotherapy (nCRT) between January 2017 and December 2020 at the Shandong Cancer Hospital and Institute were enrolled in this study. The original dose of external beam RT consisted of 1.8 Gy/day, five days/week for five weeks for a total dose of 41.4 Gy to the tumor. This was accompanied by platinum-based chemotherapy. Surgery was performed 4–8 weeks after the completion of nCRT. ESCC tissues were obtained from endoscopic biopsies at initial diagnosis and surgical resection. The use of human samples was approved by the Ethics Committee of our medical center. The patients were informed of this research and provided their written consent for the use of their specimens for research purposes. This study was performed in accordance with the principles of the Declaration of Helsinki and its later amendments.

### Mice and cell lines

C57BL/6 wild-type (WT) mice (6–8 weeks old) were purchased from Huafukang Biotechnology Co., Ltd (Beijing, China). Basic leucine zipper transcription factor ATF-like 3 deficient (BATF3^−/−^) mice were donated by Liufu Deng (Shanghai Jiao Tong University, Shanghai, China). All mice were used in accordance with the animal experimental guidelines set forth by the Institute of Animal Care and Use Committee of the Shandong Cancer Hospital and Institute. MC38, B16 F10, and LLC cell lines were purchased from the Shanghai Cell Collection, Chinese Academy of Sciences. All cell lines were authenticated using short tandem repeats profiling within the past three years. MC38 and LLC cells were cultured in 5% CO_2_ and maintained in vitro in Dulbecco’s modified Eagle medium supplemented with 10% fetal bovine serum, 100 U/ml penicillin, and 100 mg/ml streptomycin. B16-F10 cells were maintained in vitro in Roswell Park Memorial Institute 1640 medium. All cell lines were authenticated and free of mycoplasma.

### Tumor inoculation and treatment

MC38, B16 F10, or LLC tumor cells (1 × 10^6^) were injected subcutaneously into the flanks of the experimental mice. Tumor volumes (TVs) were measured according to the following formula: TV = length × width^2^ × 0.5. RT was performed using an X-ray machine (256 kV, 10 mA) with a dose rate of 1.65 Gy/min. When the tumors reached 5–6 mm in diameter, the mice were anaesthetized and irradiated with a single dose to the tumor area while the rest of the body was shielded. The RT dosage (15 Gy) was chosen according to a previous study, wherein 15 Gy combined with immunotherapy triggered a good T cell effect [17]. The TVs were measured twice per week. For the CD8 + T cell, CD4 + T cell, and NK cell depletion experiments, 250 μg of anti-CD8 (clone YTS 169.4; Bio-XCell), 200 μg of anti-CD4 (clone YTS 177; Bio-XCell, Lebanon, NH, USA), or 250 μg of anti-asialo GM1 (Lot NO. EBF6552, WAKO Pure Chemical Industries, Ltd., Richmond, VA, USA) [18] antibodies per mouse were delivered four times by intraperitoneal injection every three days, starting one day prior to RT. For the TIGIT blockade experiment, 200 μg of anti-TIGIT therapy (clone IG9; BioX Cell, West Lebanon, NH, USA) was administered intraperitoneally to the mice starting from the day of RT in the MC38 and LLC tumor models. B16 melanoma is an aggressive tumor with poor immunogenicity [19, 20] that is poorly controlled with anti-TIGIT [7]. Therefore, we designed the present experiment in reference to the previous literature and anti-TIGIT treatment was given earlier than for the MC38 and LLC tumor models (i.e., starting on day 3 after B16-F10 inoculation); TIGIT mAb was then given every three days for a total of four injections. As described previously, the FMS-like tyrosine kinase 3 ligand (Flt3L; clone Flt-3L-Ig; Bio-XCell, 10 ng/mouse/injection) was injected intraperitoneally into the mice for nine consecutive days after tumor inoculation [21].

### Flow cytometry

To obtain single-cell suspensions, tumor tissues were dissected into approximately 1–3 mm^3^ fragments and digested by 1 ug/uL collagenase IV (Sigma-Aldrich, St. Louis, MO, USA) and 0.2 ug/uL DNase I (Solarbio, Beijing, China) for 30 min at 37 °C. Cells were blocked with anti-FcR (clone 2.4G2; BD Biosciences, Franklin Lakes, NJ, USA) and then stained with antibodies against CD45, CD3, CD8, CD4, NK1.1, TIGIT, IFN-γ, TNF-*α*, CD11b, CD11c, I-AK, CD103, IL-10 and IL-12. For intracellular cytokine staining, cells were stimulated with Cell Activation Cocktail (with Brefeldin A) (Cat. 423,303; Biolegend, San Diego, CA, USA) at 37 °C for 4–5 h. After staining of surface markers, cells were fixed and permeabilized followed by staining with IFN-*γ*, TNF-*α*, IL-10, and IL-12. Fixable Viability Stain 780 (Cat. 565,388; BD Biosciences) was used for live/dead discrimination. Samples were collected using a FACSCalibur™ flow cytometer (BD Biosciences), and data were analyzed using FlowJo software (Tree Star Inc., Ashland, OR, USA).

### Multiple immunofluorescence staining

Samples obtained from biopsy or surgical resection were routinely fixed in 10% neutral buffered formalin, embedded in paraffin, and cut into 4 μm sections. The tumor tissues were then dewaxed and rehydrated. The slides were stained using an immunohistochemical technique [22] that labeled the following primary antibodies CD8 (1:800, 81254S, CST, San Antonio, TX, USA), CD4 (1:200, ab183685, Abcam, Cambridge, UK), CD56 (1:200, NBP2-38,452, Novus, St. Louis, MO), TIGIT (1:200, NBP2-79,793, Novus, St. Louis, MO), F4/80 (1:250, 12653 T, Cell Signaling Technology, Danvers, MA, USA), and CD11c (1:100, ab254183, Abcam, Cambridge, UK). For fluorescence multiplex immunohistochemistry, an OPAL™ dye kit (Cat. #OP7DS1001KT; OPAL REAGENT; PerkinElmer, Waltham, USA) was used. The experimental procedure was performed according to the manufacturer’s instructions [23]. Briefly, the slides were initially boiled in a microwave (20 min at 100 °C) for antigen retrieval. Two different primary antibodies were followed with diamidinoino-2-phenylindole (DAPI) staining within each experiment. One circle of antibody staining included peroxidase blocking, application of the primary antibody, detection with a secondary horseradish peroxidase-conjugated antibody, fluorescence dye detection, and removal of the bound antibodies by microwave treatment (20 min at 100 °C). The slides were subsequently counterstained with DAPI and mounted in an antifade solution. All slides stained with fluorescent dyes were scanned using an Akoya Biosciences automated epifluorescence microscope (Malborough, MA, USA).

### Histopathologic analysis

The percentage of residual tumor evident on microscopic examination of surgically resected specimens were evaluated by two pathologists. The Chirieac modified tumor regression grade (TRG) system was used as the reference standard [24]. Tumor response to nCRT was graded as follows: TRG 1, no residual carcinoma; TRG 2, 1–10% residual carcinoma; TRG 3, 11–50% residual carcinoma; and TRG 4, > 50% residual carcinoma.

### Statistical analysis

The experimental data were analyzed using GraphPad Prism software (v. 8.0, GraphPad Software Inc., San Diego, CA, USA). Student’s t-test or two-way analysis of variance was used to evaluate statistical differences, as appropriate. Data are represented as means ± standard errors of the mean (SEM) for all figure panels. Statistical significance was set at *p* < 0.05 and was indicated by an asterisk in the figures.

## Results

### RT upregulates TIGIT expression in tumor-infiltrating lymphocytes in ESCC patients

Although RT has consistently been shown to activate key elements of the immune system [14, 25], previous studies have found that RT is involved in the negative regulation of T cells (such as through the PD-L1/PD-1 and CD47/CD68 axes) [26, 27]. RT in combination with different forms of immunotherapy, such as anti-PD-1, and anti-CTLA4 antibodies, consistently improves local tumor control and leads to improved systemic tumor control (termed the abscopal effect) [28]; however, relapses often occur. Incomplete tumor eradication could be due to other T cell-negative regulatory pathways, such as the TIGIT/CD155 axis [16]. To better understand the potential influence of RT on TIGIT expression in tumor-infiltrating lymphocytes (TILs), we performed a post-hoc exploratory analysis that compared variations in TIGIT expression in CD8 + T cells, CD4 + T cells and NK cells (Fig. S1) between pre- and post-treatment tumor samples in each group using immunofluorescence staining. These samples were collected from ESCC patients who received nCRT.

**Fig. 1:**
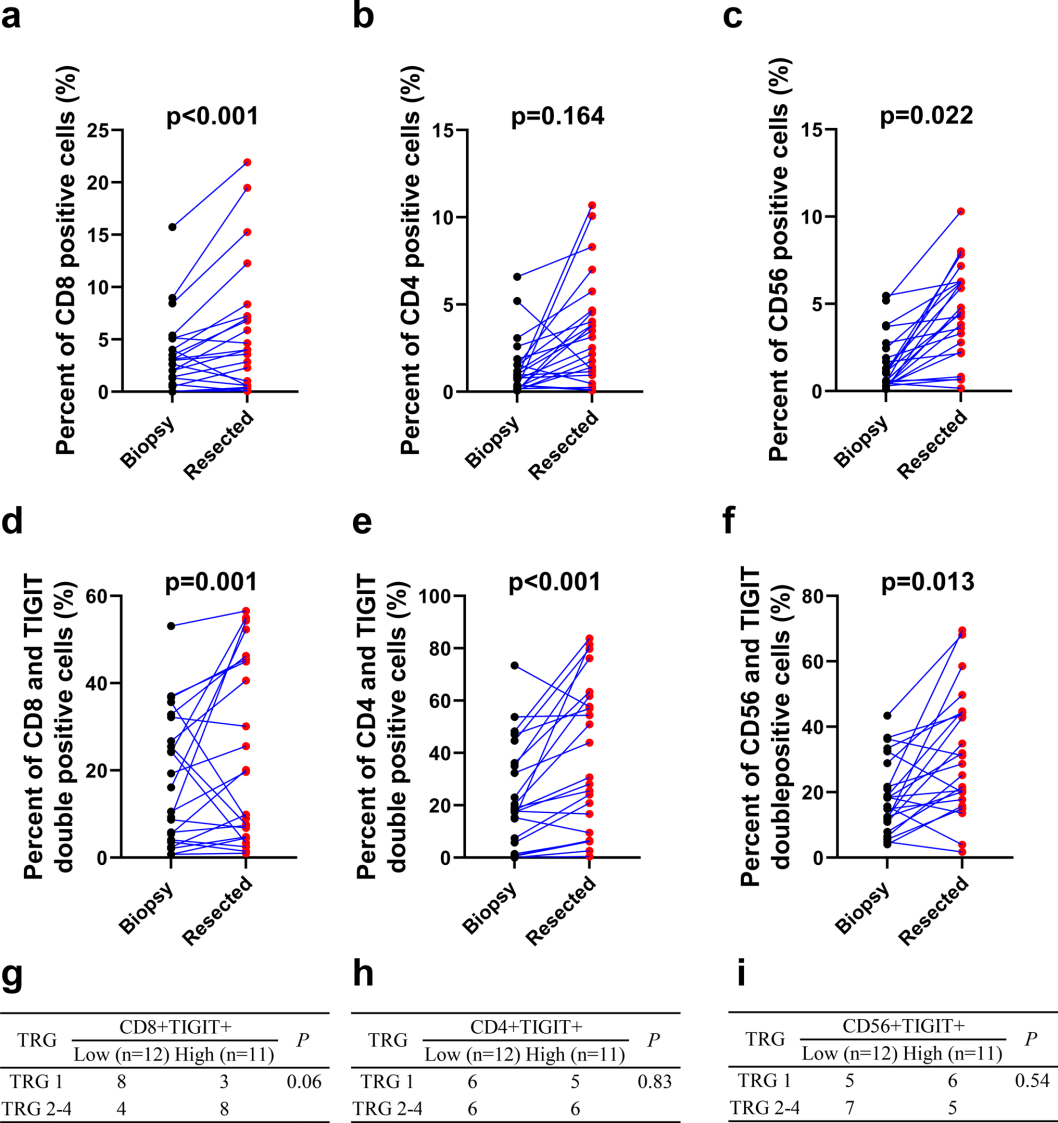
Increased TIGIT expression in tumor-infiltrating lymphocytes following RT and was statistically significantly associated with unfavorable treatment responses in ESCC patients. (**a**–**c**). CD8 + T-cells, CD4 + T-cells, and NK cells percentages in pre-and post-treatment tumor samples from ESCC patients treated with nCRT. (**d**–**f**). Percentage of TIGIT expression on CD8 + T cells, CD4 + T cells, and NK cells in pre- and post-treatment tumor samples from ESCC patients treated with nCRT. (**g**–**i**). Percentage of TIGIT + CD8 + T-cells, TIGIT + CD4 + T-cells, and TIGIT + NK cells in pre-treatment tissues and the clinicopathologic response rate after nCRT. According to the median value of TIGIT expression, cells were divided into low or high expression groups. ESCC, esophageal squamous cell carcinoma; nCRT, neoadjuvant chemoradiotherapy; NK, natural killer; RT, radiotherapy; TIGIT, T cell immunoreceptor with immunoglobulin and ITIM (immunoreceptor tyrosine-based inhibitory motif) domains

Twenty-three patients with ESCC were included in this study. Based on automated image analysis using an automated epifluorescence microscope, we found a statistically significant increase in the number of CD8 + T and NK cells, except for CD4 + T cells (Fig. 1a–c) in post nCRT tumor samples. CD8 + T, CD4 + T, and NK cell infiltration levels in tumor tissue prior to nCRT were 3.5% ± 3.6%, 1.4% ± 1.6 and 1.6% ± 1.6%, respectively; after nCRT, these values were increased to 5.6% ± 6.1%, 3.5% ± 3.0 and 4.5% ± 2.6%, respectively (*p* < 0.001, *p* = 0.164 and *p* = 0.022). These results indicate that tumor tissue lymphocyte infiltration was statistically significantly increased after nCRT. However, TIGIT expression on CD8 + T cells, CD4 + T cells and NK cells was also elevated after nCRT (pre-nCRT value vs. post-nCRT value, *p* < 0.001, 18.2% ± 14.6% vs. 23.9% ± 20.2%%; *p* = 0.022, 23.2% ± 19.6% vs. 38.6% ± 27.5% and 18.9% ± 11.1% vs. 31.5% ± 1 8.3%, respectively) (Fig. 1d–f). To determine whether TIGIT expression on TILs influences the response to nCRT, we compared TRG with pre-treatment TIGIT expression in patients treated with nCRT. Patients with a score of TRG 1 after nCRT tended to have lower levels of TIGIT + CD8 + T cells in their pre-treatment samples (*p* = 0.06, Fig. 1g). However, TIGIT + CD4 + T cells and TIGIT + CD56 + NK cells rates in pre-treatment tumor were not statistically significantly correlated with TRG (*p* > 0.05, Fig. 1h–i).

We then classified patients according to the time from nCRT to surgery (4 or 8 weeks) and found that the expression of TIGIT + CD8 + T cells increased statistically significantly at four weeks after nCRT as compared with at eight weeks after nCRT (i.e., a 4.55 ± 7.72 vs. a 1.92 ± 1.83 fold change). These data suggest that nCRT can increase lymphocytes infiltration in tumors, but that these lymphocytes are more likely to present with an immunosuppressive phenotype; this may reduce the effectiveness of RT.

### RT upregulates TIGIT expression in TILs in a tumor-bearing mouse model

**Fig. 2:**
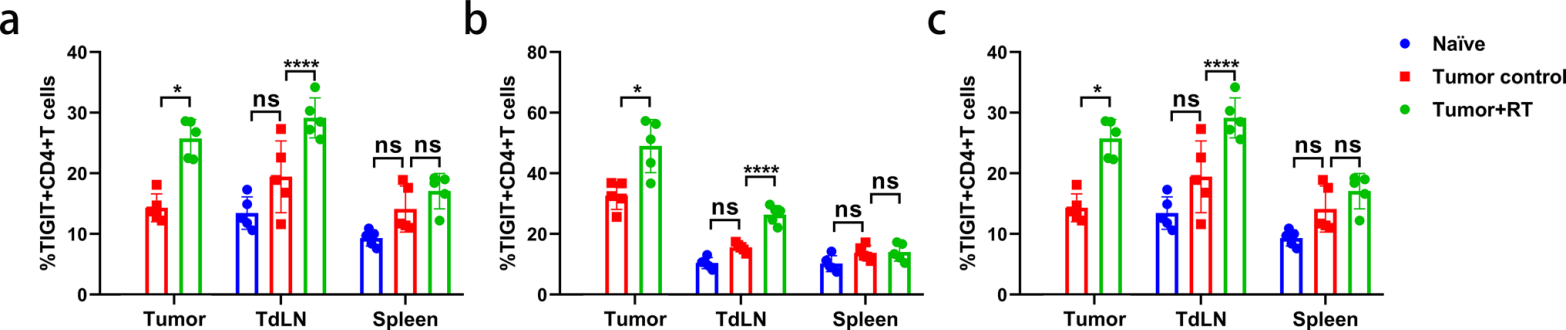
TIGIT expression was upregulated in tumor-infiltrating lymphocytes and TdLNs after RT in a mouse model. **a.** CD8 + T cells had significantly higher expression of TIGIT in tumor and TdLN samples after RT. (**b-c**). CD4 + T-cells and NK cells also had increased TIGIT expression after RT in tumor and TdLN samples compared to those that did not receive RT. The spleen samples were unchanged. The gating strategy was shown in Fig. S2. *p < 0.05; ***p < 0.001; ****p < 0.0001. NS, not statistically significant; RT, radiotherapy; TdLNs, tumor draining lymph nodes; TIGIT, T cell immunoreceptor with immunoglobulin and ITIM (immunoreceptor tyrosine-based inhibitory motif) domains

While elevated TIGIT expression has been demonstrated in human ESCC tissue samples after receiving nCRT, whether RT alone can also affect the expression of TIGIT on lymphocytes whether targeting the TIGIT/CD155 pathway enhances the efficacy of RT, and the mechanisms that underlie these phenomena remain unknown. To investigate the potential synergistic benefits of combination therapy, we selected naïve and the MC38 tumor-bearing mouse model for further experimentation. We first sought to determine whether TIGIT expression in lymphocytes was elevated after RT in tumor-bearing mice. Untreated and treated mice were sacrificed on day 10 after receiving 15 Gy. Tumors, tumor-draining lymph nodes (TdLNs), and spleens were harvested. Immune cells were isolated and flow cytometry was used to identify CD8 + T cell, CD4 + T cell, and NK cell populations. The gating strategy is shown in the Supplementary Material (Fig. S2). First, we found that TIGIT expression in tumor lymph nodes as well as in the spleens of tumor-bearing mice was up-regulated compared with that in non-tumor-bearing mice, although no statistically difference was observed. Ten days after RT, increased expression of TIGIT was observed in CD8 + T cells, CD4 + T cells, and NK cells as compared with expression levels in the same cell populations of non-irradiated control tumors (Fig. 2a–c). TIGIT expression was statistically significantly increased in CD8 + T cells, CD4 + T cells, and NK cells in TdLNs (*p *< 0.05). TIGIT expression did not statistically significantly change in CD8 + T, CD4 + T, and NK cells in the spleen after RT (*p* > 0.05; Fig. 2a–c). We also found that the expression levels of TIGIT on CD8 + T cells, CD4 + T cells and NK cells in TILs and TDLNs treated with RT were statistically significantly higher than expression levels on cells not treated with RT in LLC and B16 tumor-bearing mouse models. However, there was no statistically significant difference in the expression of TIGIT in spleen lymphocytes after RT (Fig. S3). These data raise the possibility that overcoming tumor-acquired radioresistance by blocking the TIGIT/CD155 axis is may be a viable treatment strategy.

### Anti-TIGIT therapy enhances the anti-tumor effect of RT

**Fig. 3:**
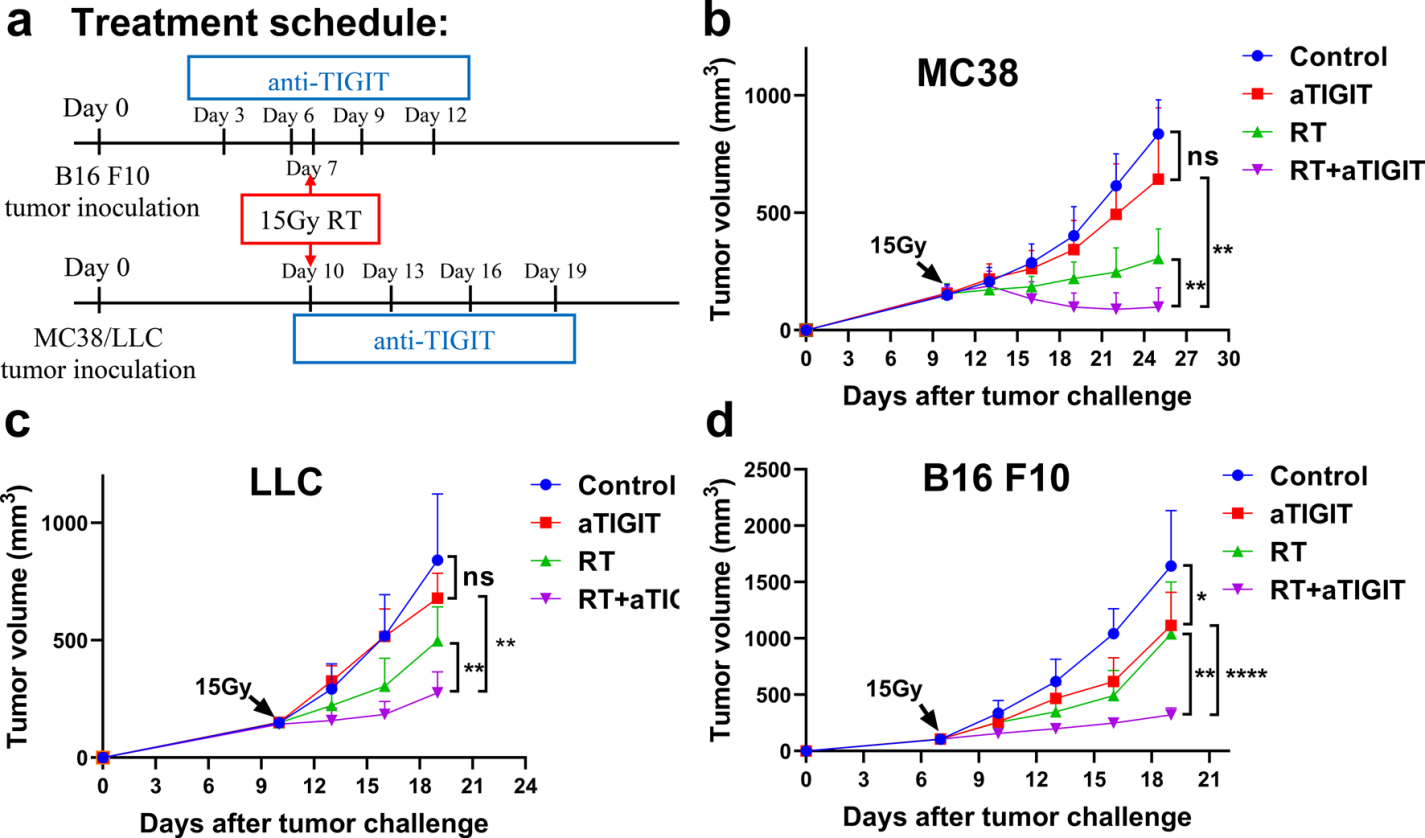
RT and anti-TIGIT therapy showed synergistic anti-tumor effects in MC38, LLC and B16 F10 mouse models. **A.** Diagram depicting the experimental set up including treatment schedules. C57BL/6 mice were inoculated subcutaneously on day 0 with 1 × 10.^6^ MC38, LLC or B16 F10 cells. MC38 and LLC tumor-bearing mice (*n* = 5–7mice/group) were treated with one 15 Gy dose 10 days post-tumor inoculation. Mice received 200 ug anti-TIGIT (clone IG9) or isotype IgG intraperitoneally every three days, for a total of four injections. **b**. Combination therapy greatly delayed MC38 tumor growth compared with individual treatments. **c**. Combination therapy greatly delayed LLC tumor growth compared with individual treatments. **d**. Combination therapy greatly delayed B16 F10 tumor growth compared with individual treatments. Anti-TIGIT therapy alone inhibited tumor growth compared with the controls. *p < 0.05; **p < 0.01; ****p < 0.0001. IgG, immunoglobulin G; NS, not statistically significant; RT, radiotherapy; TIGIT, T cell immunoreceptor with immunoglobulin and ITIM (immunoreceptor tyrosine-based inhibitory motif) domains

TIGIT expression in the tumor microenvironment (TME) has previously been associated with poor outcomes following chemoradiotherapy in patients with cancer [29, 30]. We also found that TIGIT expression in CD8 + T cells, CD4 + T cells and NK cells increased with RT. Therefore, we hypothesized that anti-TIGIT therapy could enhance immune-mediated responses to RT and synergistically boost anti-tumor effects. To test our hypothesis, MC38, LLC, and B16 F10 cells were implanted in WT C57BL/6 mice; 7–10 days later, tumors were treated with RT (15 Gy), anti-TIGIT alone, or RT plus anti-TIGIT (Fig. 3a). We found that anti-TIGIT therapy alone had no statistically significant impact on tumor growth (except for the B16 F10 model), whereas RT alone slowed tumor progression. Treatment with a combination of RT and anti-TIGIT therapy effectively controlled tumor growth in the MC38 tumor model (Fig. 3b, Fig. S4). The effectiveness of combination treatment was also confirmed in the poorly immunogenic LLC lung cancer model (Fig. 3c). Anti-TIGIT therapy alone could inhibited tumor growth only in the melanoma B16 F10 model. Treatment with a combination of RT + anti-TIGIT also effectively controlled tumor growth in this model (Fig. 3d). These results suggest that anti-TIGIT treatment combined with RT improves primary tumor control.

### The addition of TIGIT inhibitor to RT enhances anti-tumor effects

**Fig. 4:**
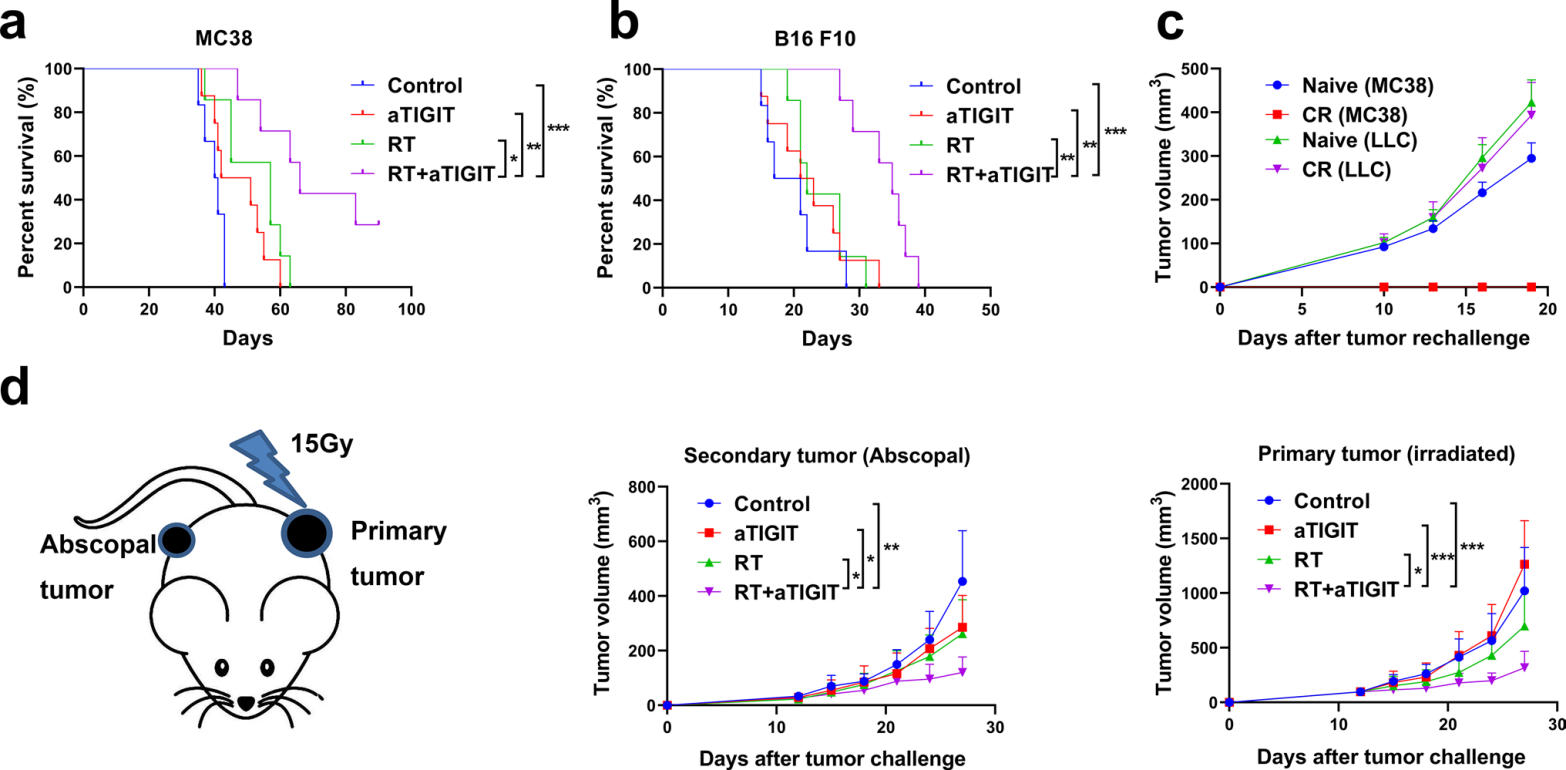
RT and anti-TIGIT combination therapy improve long-term survival immune memory effect, and abscopal effect. (**a**-**b)**. The Kaplan Meier survival curve depicts the primary endpoint in mouse models of MC38 and B16 F10 treated with anti-TIGIT therapy, RT, and RT + anti-TIGIT therapy, respectively. **c**. Ninety days after tumor eradication, the mice were rechallenged with 2 × 10.^6^ MC38 cells on the opposite flank. Both groups did not receive additional treatment. Tumor-free mice that underwent combination therapy were resistant to the tumor rechallenge. **d**. Combination treatment greatly reduced the growth of secondary tumors. Tumors on the right flank were measured and monitored. Representative data are shown from two experiments conducted with five mice per group. Data are presented as means ± SEM (standard errors of the mean). *p < 0.05; **p < 0.01; ***p < 0.001. RT, radiotherapy; TIGIT, T cell immunoreceptor with immunoglobulin and ITIM (immunoreceptor tyrosine-based inhibitory motif) domains

Next, we assessed whether the improved anti-tumor response in turn improved survival, protective immunity and systemic tumor control. MC38 and B16 F10 cells were implanted in the C57BL/6 mice, and the tumors were treated as shown in Fig. 3a. For the MC38 tumor model, treatment with RT plus anti-TIGIT therapy was effective in extending mouse survival time compared to the control group (p < 0.001, median survival 66 vs. 40.5 days) (Fig. 4a). For the B16 F10 tumor model, treatment with RT plus anti-TIGIT therapy was also effective in extending mouse survival time compared to the control group (*p* = 0.001, median survival 35 vs. 19 days) (Fig. 4b). In the LLC tumor model, mice under RT + TIGIT treatment lived statistically significant longer than those in the other three treatment groups (Fig S5). These results confirm that RT plus anti-TIGIT therapy can prolong the survival time of tumor-bearing mice.

Among the three tumor models (Fig. 3), only a portion of the MC38 tumors model achieved complete response (CR) in combination therapy. Thus, mice that previously cleared MC38 tumors were rechallenged with much higher doses (2 × 10^6^ cells) of MC38 tumor cells on the opposite flank and of LLC (1 × 10^6^ cells) in their left thoracic flanks at three months after complete tumor rejection, in order to examine whether tumor antigen specific immunity was established. No palpable MC38 tumors were detected in the treated mice after a few weeks, whereas tumors in naive mice were palpable after seven days. Mice that previously cleared MC38 tumors did not mount a protective antitumor response to novel LLC tumors (Fig. 4c, Fig. S6). These data indicate the induction of tumor antigen specific immunity in the treated mice.

Other studies have shown that RT combined with immunotherapy not only controlled local irradiated tumors but also controlled distant non-irradiated tumors (secondary tumors) [31]. Thus, we tested whether combination treatment with RT and anti-TIGIT therapy could also exert abscopal effects on metastases that did not receiving RT. To test this, MC38 cells were implanted in both flanks of the C57BL/6 mice. Tumors intended for local RT treatment received five times more tumor cells than the contralateral flank, which served as the secondary tumors (1 × 10^6^ cells vs. 0.2 × 10^6^ cells) in the current study. Primary tumors that received treatment and the therapies used herein are shown in Fig. 3a. Growth delay in secondary tumors was observed in the RT plus anti-TIGIT group, but not in groups that received either treatment alone (*p* = 0.002 vs. control; *p* = 0.011 vs. anti-TIGIT alone; *p* = 0.029 vs. RT alone; Fig. 4d). These results suggest that anti-TIGIT treatment not only improves the effects of RT on primary tumors, but can also control secondary tumors (abscopal effects).

### The synergistic effect of RT and anti-TIGIT combination therapy is dependent on CD8 + T cells

**Fig. 5:**
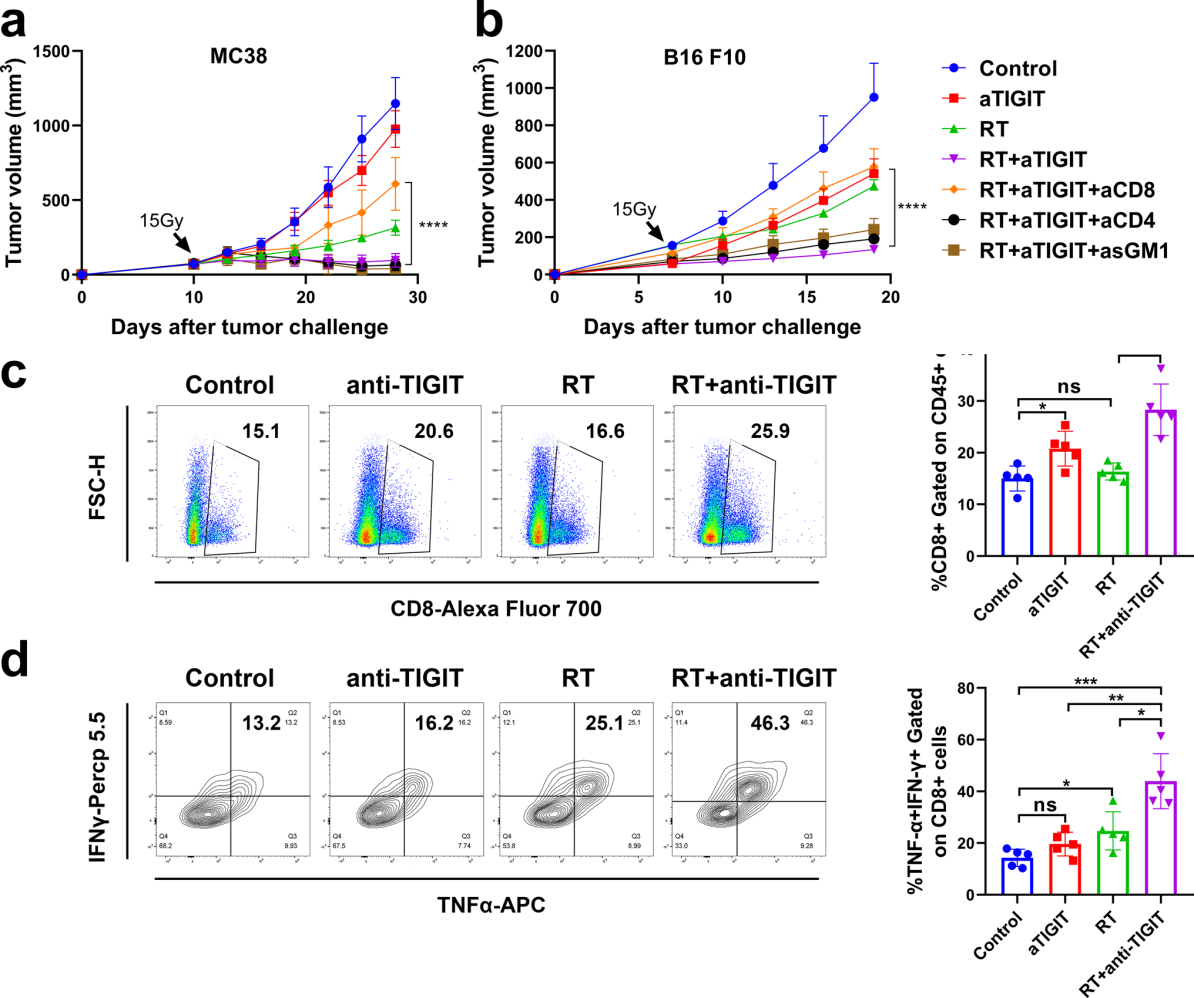
CD8 + T cells are required for effective RT and anti-TIGIT combination treatment. a. Tumors (MC38) received 15 Gy and mice were treated with anti-TIGIT therapy, as described in Fig. 3a. Starting from 1 day before RT, 250 μg of depletion antibodies against CD8 + T, CD4 + T, and NK cells were injected intraperitoneally every 3 days for a total of four injections. b. Tumors (B16 F10) received 15 Gy and mice were treated with anti-TIGIT therapy, as described in Fig. 3a. Depletion antibodies against CD8 + T cells, CD4 + T cells and NK cells were used. Representative data are shown from three **a** and two **b** experiments conducted with 5–6 mice per group. **c**. Combination therapy greatly enhanced the antigen-specific response of CD8 + T cells. d. Representative flow cytometry plots of IFN-*γ* and TNF-*α* expression on the CD8 + T cells extracted from the TILs of the control, anti-TIGIT alone, RT alone, and RT plus anti-TIGIT treatment groups (left). For intracellular cytokine staining, cells were stimulated with Cell Activation Cocktail (with Brefeldin A) (1:500) for 4–5 h before being harvested for cell surface staining, after which cells were fixed and permeabilized and stained with IFN-*γ* (interferon gamma) and TNF-*α* (tumor necrosis factor alpha). The gating strategy was shown in Fig S4. Quantification of IFN-γ/TNF-*α* dual production is shown on the right. Data are represented means ± SD (standard deviations) with two independent biological duplications. *p < 0.05; **p < 0.01; ***p < 0.001; ****p < 0.0001. NK, natural killer; NS, not statistically significant; RT, radiotherapy; TIGIT, T cell immunoreceptor with immunoglobulin and ITIM (immunoreceptor tyrosine-based inhibitory motif) domains; TILs, tumor infiltrating lymphocytes

TIGIT expression has been demonstrated to be tightly restricted to lymphocytes, mainly on T cell subsets (including regulatory and memory T cells) and NK cells [4, 32]. To investigate the importance of CD8 + T cells, CD4 + T cells, and NK cells in combination therapy, CD8 + T, CD4 + T, and NK cells were depleted using antibodies in mice treated with RT plus anti-TIGIT therapy. In the MC38 model, depletion of CD8 + T cells completely abolished the effectiveness of the combination treatment, resulting in rapid tumor growth. However, depletion of CD4 + T cells and NK cells had no statistically significant effect on the effectiveness of combination treatment (*p* < 0.001, Fig. 5a). We also observed the same tumor growth trends as in the B16 F10 and LLC tumor model (*p* = 0.0001, Fig. 5b, Fig. S7). Altogether, these results demonstrate that CD8 + T cells play a major role in the therapeutic effects of RT combined with anti-TIGIT therapy.

Next, we hypothesized that the combination of RT and anti-TIGIT treatment could further improve T cell function. Ten days after RT, tumors from tumor-bearing mice were removed, and the numbers and functional factors (IFN-γ [interferon gamma]and TNF-*α* [tumor necrosis factor alpha]) of CD8 + T cells were analyzed using flow cytometry (gating strategy: Fig. S8). The number of CD8 + T cells was statistically significantly increased in the TILs of mice that received combination treatment as compared to those that received RT or anti-TIGIT therapy alone (*p* = 0.0015, anti-TIGIT vs. RT + anti-TIGIT; *p* < 0.0001, RT vs. RT + anti-TIGIT) (Fig. 5c). We also observed a higher frequency of tumor-infiltrating CD8 + T cells expressing TNF-*α* and IFN-γ in the combination treatment group than in the individual anti-TIGIT or RT groups (Fig. 5d). In addition, RT combined with anti-TIGIT treatment resulted in a statistically significant higher production of TNF-*α* and IFN-*γ* in CD8 + T cells (Fig. S9). In the secondary tumors, we also found a higher frequency of tumor-infiltrating CD8 + T cells in the TILs and a higher frequency of CD8 + T cells expressing TNF-*α* and IFN-*γ* in the combination treatment group than in the individual anti-TIGIT or RT groups (data not shown). Our results indicate that RT plus anti-TIGIT therapy improves tumor control by enhancing CD8 + T cell function in the TME and by enhancing systemic activation of tumor-specific T cells in distant tumors.

### RT and anti-TIGIT combination therapy increase DC accumulation at the tumor site

**Fig. 6:**
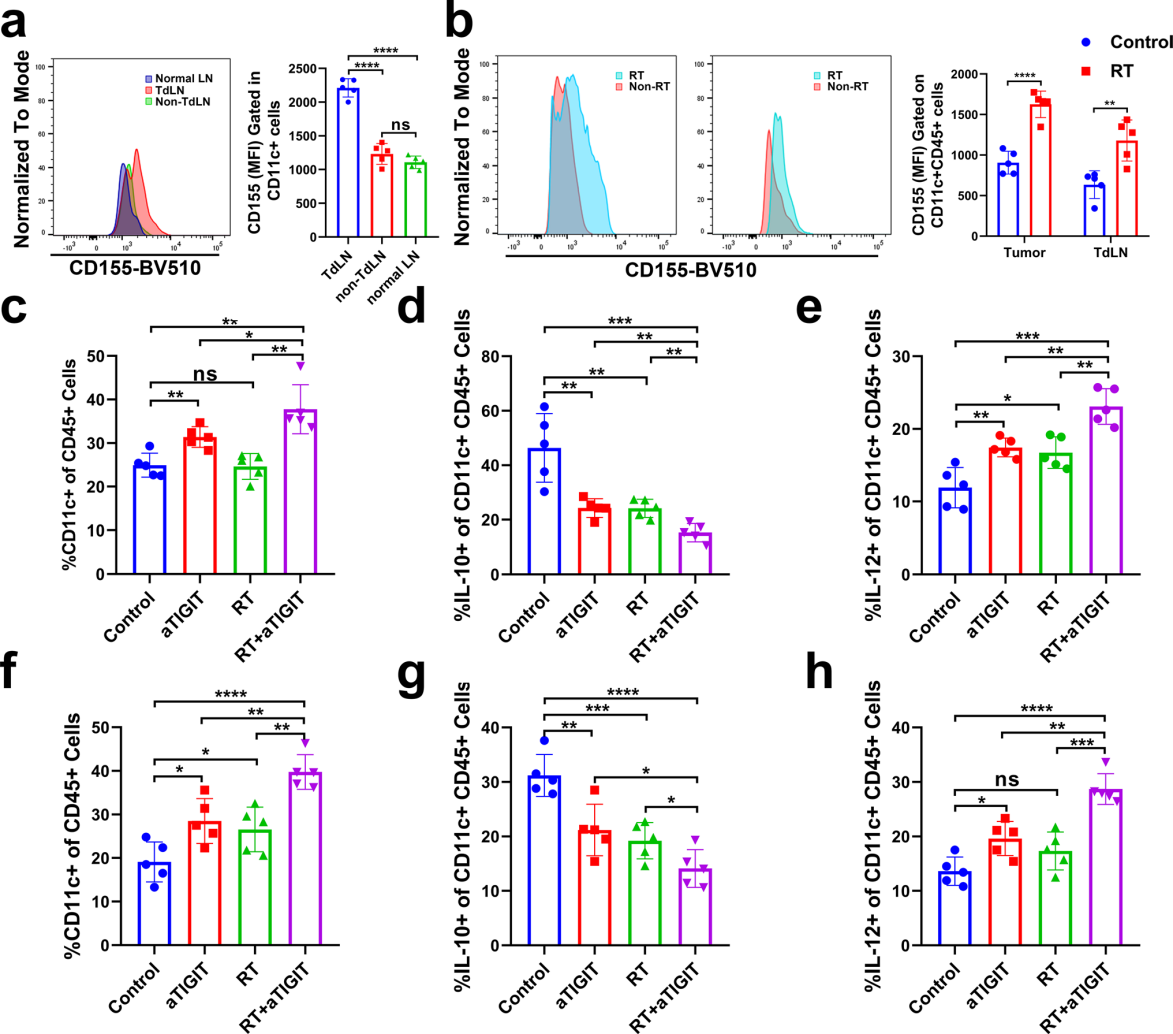
The addition of a TIGIT inhibitor to RT enhances DC numbers and function in MC38 tumor-bearing mice. Mice were inoculated with MC38 cells on day 0. On day 10, mice were given either isotype IgG, anti-TIGIT therapy, RT, or RT plus anti-TIGIT therapy. Tumors and TdLNs were excised on day 10. **a**. CD155 expression in CD11c + DCs purified from MC38 TdLNs (red lines), non-draining LNs (green lines), and healthy mouse LNs (purple lines). Representative flow cytometry histogram (left) and quantification of CD155 mean fluorescent intensity in DCs (right). **b**. Representative flow cytometry contour plots of CD155 expression on DCs after RT in tumors and TdLNs (*n* = 5); Quantitation of CD155 MFI on DCs is shown on the right. **c**. Bar graphs show the DC density in tumor tissues as measured by flow cytometry. (**d**-**e)**. Intracellular IL-10 and IL-12 (interleukin 10 and 12) levels on gated CD11c + DCs from tumor tissues. *f*. Bar graphs show the DC density in TdLNs as measured by flow cytometry. (**g**-**h**). Intracellular IL-10 and IL-12 levels on gated CD11c + DCs from TdLNs. For intracellular cytokine staining, cells were stimulated with Cell Activation Cocktail (with Brefeldin A) (1:500) for 4–5 h before being harvested for cell surface staining, after which cells were fixed and permeabilized and stained with IL-10, and IL-12. Results are shown as the means ± SEM (standard errors of the mean) for one experiment (n = 5). *p < 0.05; **p < 0.01; ***p < 0.001; ****p < 0.0001. DCs, dendritic cells; IgG, immunoglobulin G; LNs, lymph nodes; NS, not statistically significant; RT, radiotherapy; TdLNs, tumor draining lymph nodes; TIGIT, T cell immunoreceptor with immunoglobulin and ITIM (immunoreceptor tyrosine-based inhibitory motif) domains

A previous study found that efficient antitumor immunity often requires cooperation between DCs and T cells [33]. DCs often have superior antigen cross-presentation capabilities that are activated by local RT [34], thereby leading to stronger CD8 + T cell immunity [35, 36]. Many strategies have been developed to target DCs in cancer, such as the generation of DC-based vaccines, as well as the administration of antigens with immunoregulators that mobilize and activate DCs [37–39]. Another approach to improve DCs function is to overcome the immunosuppressive activities of cancer-associated DCs [40, 41]. TIGIT is expressed on T cells and NK cells that bind to CD155 on the DC surface, which drives DCs toward a more immune tolerant phenotype [42, 43]. Thus, we hypothesized that the potential interaction of TIGIT/CD155 on cancer-associated DCs might be important in the regulation of immune function in cancer. To determine the role of DCs in RT combined with anti-TIGIT therapy, we first measured the expression of CD155 on DCs in TdLNs and non-TdLNs from a tumor mouse model and normal-LNs from healthy mice using flow cytometry. DCs expressed CD155 at higher levels in TdLNs, as compared to DCs present in the non-TdLNs and normal-LNs (Fig. 6a, Fig. S10). Additionally, an increase in CD155 expression was observed in DCs after RT as compared with the same cell populations in non-irradiated control tumors (Fig. 6b), prompting us to examine whether TIGIT blockade increases the ability of DCs to promote proliferation or activate CD8 + T cells in TdLNs and tumor tissues in order to enhance anti-tumor immunity. Next, anti-TIGIT therapy and RT were administered to MC38 tumor-bearing mice starting on day 10 post-tumor inoculation, and tumors and TdLNs were analyzed on day 18. RT plus anti-TIGIT blockade led to a statistically significant increase in the total number of DCs present in tumor tissues and TdLNs (Fig. 6c, f, Fig. S11a). It has also been reported that increased CD155 interaction with TIGIT promotes the expression of IL-10 in DCs and reduces their antigen-presenting capacity to CD8 + T cells [44], and that IL-12 produced by antigen-presenting cells can regulate the activation and differentiation of lymphocytes [45]. We analyzed whether there were changes in the production of IL-10 and IL-12 in the DCs of tumor tissues and TdLNs in relation to combination therapy. TIGIT blockade alone or in combination with RT statistically significant enhanced the production of IL-12 and inhibited the production of IL-10 in DCs (Fig. 6d–e, g–h). These results indicate that RT combined with anti-TIGIT therapy may activate immune responses in part by increasing the infiltration of DCs and the production of cytokines in tumor tissues and TdLNs.

### CD103 + DCs are required for effective anti-tumor immunity responses in anti-TIGIT therapy within irradiated tumors

**Fig. 7:**
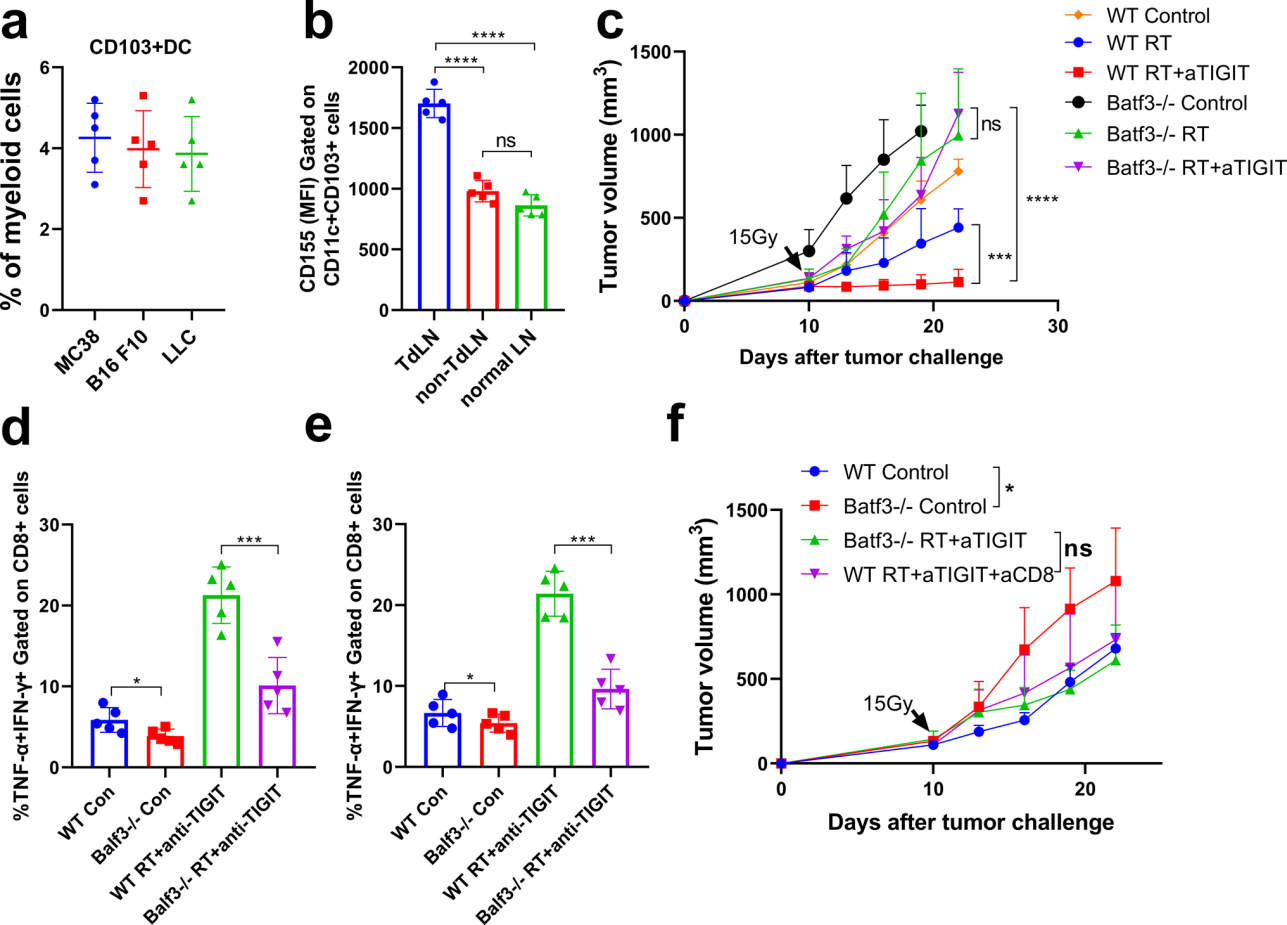
CD103 + DCs are critical for effective anti-tumor responses to RT combined with anti-TIGIT therapy. **a**. Frequency of CD103 + DCs among total myeloid cells infiltrating MC38, B16-F10, and LLC tumor models. Myeloid cells were gated on CD11b + and/or CD11c + cells within CD45 + cells. **b**. Quantification of CD155 on CD103 + DCs purified from MC38 TdLNs, non-draining LNs, and healthy mouse LNs on day 10 following tumor challenge. The gating strategy is shown in Fig. S9. **c**. MC38 tumor-bearing WT or BATF3^−/−^ mice were treated with RT plus anti-TIGIT therapy or RT alone on day 10 after the tumor challenge. Data are shown as mean ± SEMs (standard errors of the mean) for two independent experiments (*n* = 4–5). (**d**-**e**). The production of IFN-*γ* (interferon gamma) and TNF-*α* (tumor necrosis factor alpha) by CD8 + T-cells in tumor tissues **d** and TdLNs **e** was analyzed herein. f. WT and BATF3.^−/−^ mice were inoculated with MC38 cells and treated with RT and anti-TIGIT therapy as described in Fig. 3a. Moreover, 200 μg of anti-CD8 monoclonal antibodies (mAb) was administered as described in Fig. 5a. Tumor growth was monitored after RT. Data are shown as means ± SEM (standard errors of the mean) of two independent experiments (*n* = 5). *p < 0.05; ***p < 0.001; ****p < 0.0001. BATF3, basic leucine zipper transcription factor ATF-like 3; DCs, dendritic cells; LNs, lymph nodes; mAb, monoclonal antibodies; NS, not statistically significant; RT, radiotherapy; TdLNs, tumor draining lymph nodes; TIGIT, T cell immunoreceptor with immunoglobulin and ITIM (immunoreceptor tyrosine-based inhibitory motif) domains; WT, wild type

It has been reported that CD103 + DCs are the only intratumoral myeloid cells that can transport integrated antigens to TdLNs [36]. In particular, recent studies have further confirmed the key role of CD103 + DCs in the priming and effector phase of the anti-tumor T cell response and in tumor progression [46, 47]. Therefore, we evaluated the CD103 + DCs content in three tumor models. In our study, CD103 + DCs accounted for a minority of the cell population, reaching less than 5% of myeloid cells (Fig. 7a), which is consistent with the finds of a previous report [36]. In the MC38 tumor model, CD103 + DCs expressed CD155 at high levels in TdLNs, as compared to non-TdLNs and normal-LNs (Fig. 7b). Therefore, we hypothesized that CD103 + DCs would play a key role in RT plus anti-TIGIT combination therapy. To investigate the contribution of CD103 + DCs toward the effects of combination therapy, we compared MC38 tumor growth upon TIGIT blockade plus RT in WT or BATF3^−/−^ mice, which lack CD103 + CD8 + DCs [35]. Despite the high expression of CD155 in MC38 tumor cells [48], the moderate but statistically significant anti-tumor effect mediated by combination therapy was lost in the BATF3^−/−^ mice (Fig. 7c). RT plus anti-TIGIT combination therapy increased the number of CD8 + T cells and the percentage of IFNγ + TNFα + CD8 + T cells in tumor tissues and TdLNs in WT mice (Fig. 7d–e). The anti-tumor effect of combination therapy was abrogated when CD8 + T cells were not present, with tumor control being similar to that in the BATF3^−/−^ group (Fig. 7f). These results established that BATF3 lineage CD103 + DCs are necessary for combination therapy-mediated recruitment of effector CD8 + T cells within the TME.

### The Flt3 ligand dramatically expands CD103 + DC progenitors and enhances tumor control of TIGIT blockade combined with RT

**Fig. 8:**
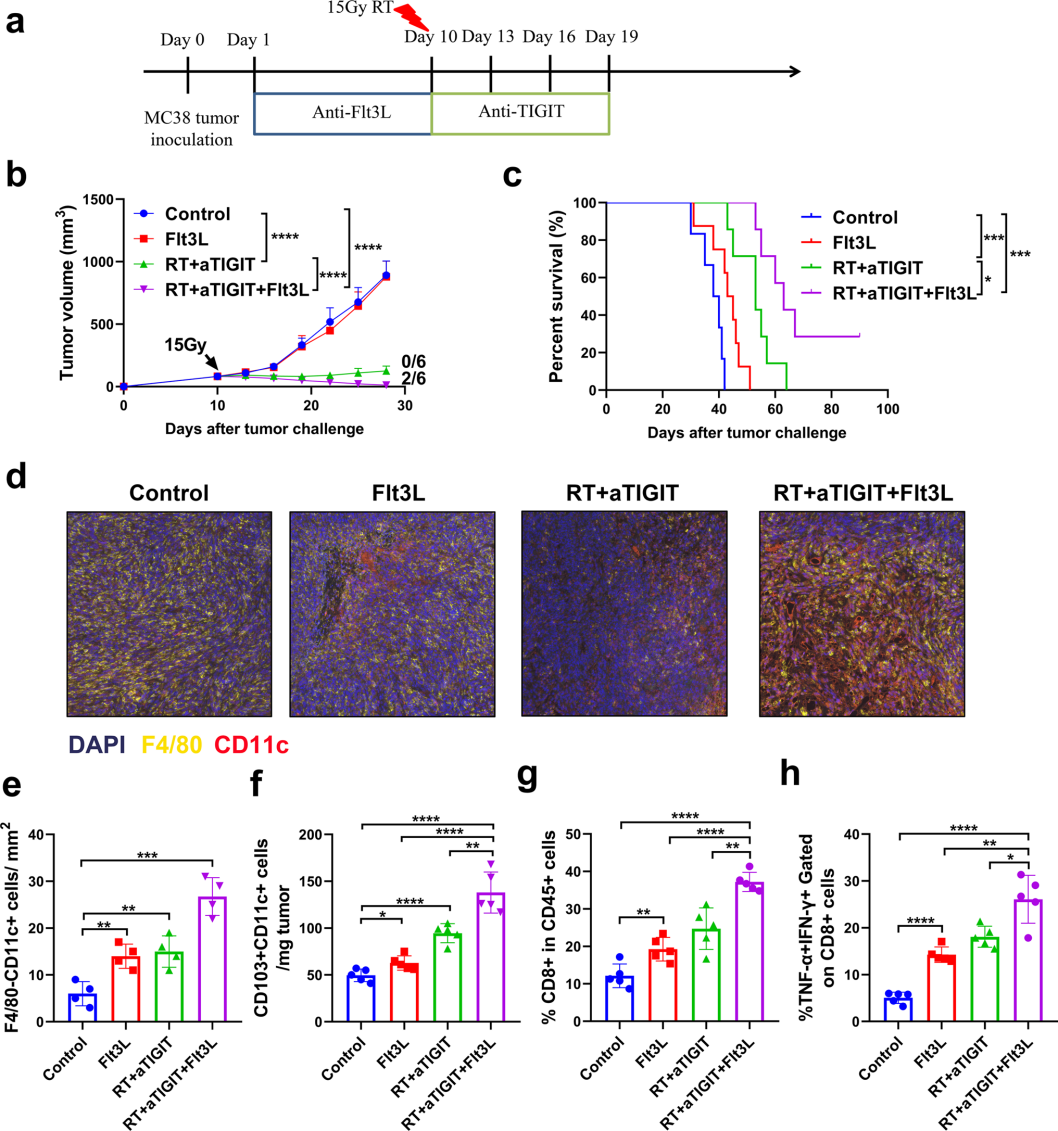
Flt3L injections improved the response of WT mice to treatment with RT and TIGIT mAb. **a**. WT MC38 tumor-bearing mice (*n* = 5 mice/group) were treated with local tumor RT in a single 15 Gy dose given on day 10. The mice received anti-TIGIT mAb on days 10, 13, 16, and 19. Some mice received Flt3L (10 ng/mouse/injection) on days 1–9 post tumor inoculation, which was injected intraperitoneally for nine consecutive days after tumor inoculation. **b**. Tumor growth is shown for each group. The data are presented as mean tumor growth ± SEM (standard errors of the mean) of two independent experiments (*n* = 5). Fractions indicate the number of mice showing complete tumor regression over the total in each group. **c**. Survival times were followed until day 90 for each group. Data shown are from one of two independent experiments performed with similar results. (**d**-**e**). Representative multiple immunofluorescence images of DCs infiltrating MC38 tumors treated with nine daily injections of Flt3L (10 ng/mouse/injection), which was injected intraperitoneally into the mice for nine consecutive days after tumor inoculation or isotype IgG starting on day 1 after tumor inoculation. Tumor biopsies were performed on day 18 after the tumor inoculation, DCs were identified as F4/80-CD11c + cells. **f**. The absolute number of CD103 + /CD11c + DCs standardized to tumor weight. **g**. On day 18, the mice were sacrificed and the percentages of CD8 + cells among the CD45 + cells were analyzed using flow cytometry. **h**. The production of IFN-*γ* (interferon gamma) and TNF-*α* (tumor necrosis factor alpha) by CD8 + T-cells was also analyzed. See Fig. S2 for the gating strategy. Data are shown as means ± SEM (standard errors of the mean) for two independent experiments (*n* = 4–5). *p < 0.05, **p < 0.01, ***p < 0.001, ****p < 0.0001. DCs, dendritic cells; IgG, immunoglobulin G; FLT3L, factor FMS-like tyrosine kinase 3 ligand; mAb, monoclonal antibody; RT, radiotherapy; TIGIT, T cell immunoreceptor with immunoglobulin and ITIM (immunoreceptor tyrosine-based inhibitory motif) domains; WT, wild type

We determined that the lack of CD103 + DCs at the tumor site limited the function of tumor-specific CD8 + T cells and therefore restricted the anti-tumor effects of combination therapy. Therefore, we injected mice with the growth factor Flt3L, a cytokine that promotes hematopoietic progenitor commitment to the DC lineage, DC survival, and proliferation in tissues in order to expand CD103 + DCs [36, 49]. Next, we explored whether the enhanced anti-tumor responses to RT plus anti-TIGIT combination therapy were changed by the addition of Flt3L. To this end, WT MC38 tumor-bearing mice were treated with RT plus anti-TIGIT in the presence or absence of Flt3L (Fig. 8a). Treatment with combination therapy was effective at controlling MC38 tumor and extending mouse survival at the level of statistical significance when compared to the control group (median survival 53 vs. 39 days, *p* < 0.001) (Fig. 8b–c). These effects were also observed in LLC tumor-bearing mice treated with RT combination therapy (i.e., blocking antibodies against TIGIT) and in mice inoculated with B16 F10 (Fig. S12a-b). However, no complete regression was reached in LLC and B16 tumor models, and all mice eventually died of tumor progression. Flt3L blockade did not have any effect on tumor growth or survival when administered alone, but tumor progression was controlled (*p* < 0.05) and survival was prolonged (*p* < 0.05) when combined with RT and anti-TIGIT treatment (Fig. 8b–c). Complete regression was observed in 33.3% (2/6) in the MC38 tumor model, and these mice survived long-term and rejected the MC38 cell rechallenge 90 days after the initial inoculation (data not shown). We found that Flt3L injections dramatically increased F4/80-CD11c + DCs and CD103 + CD11c + DCs at the tumor site, and tumor-infiltrating DCs were further increased in the combination therapy group (Fig. 8d–f). Interestingly, Flt3L treatment statistically significantly increased the intratumoral levels of CD8 + T cells as well as the production of TNFα + IFNγ + CD8 + T cells (Fig. 8g–h).

## Discussion

Our findings provide an effective therapeutic model of RT combined with anti-TIGIT therapy in achieving synergistic antitumor effects and, to the best of our knowledge, present the first study to explore the potential mechanisms underlying the interaction of these therapeutic modalities. We used multiple immunofluorescence techniques to show that RT could statistically significantly upregulate TIGIT expression on CD8 + T cells, CD4 + T cells, and NK cell surfaces in the TME of ESCC patients receiving nCRT. Similar results were observed in the MC38 tumor-bearing mouse model. Additionally, we found that higher intratumoral TIGIT + CD8 + T cell numbers were associated with lower response rates to nCRT, which is likely because this population of TIGIT + CD8 + T cells shows a high immunosuppressive phenotype [10, 12]. Therefore, our finds provide a valuable reference for evaluating the efficacy of nCRT in patients. With this in mind, we sought to understand the mechanisms of RT plus anti-TIGIT combination therapy using a mouse tumor model. In the BATF3^−/−^ tumor-bearing mice, we showed that CD103 + DCs were required to suppress tumor growth and establish antigen-specific protection against tumor rechallenge in combination therapy. This result further demonstrated the importance of CD103 + DCs in the immune cycle [36, 46]. Taken together, these results identified the functional link between TIGIT/CD155 signaling and DCs in combination therapy and demonstrated that the combination of RT and anti-TIGIT therapy can enhance host anti-tumor immune responses.

Several studies have found that anti-TIGIT therapy alone achieved good efficacy in a few tumor models [7, 10] Pre-clinical and clinical trials have also found that anti-TIGIT therapy combined with anti-PD1 may obtain a certain curative effect, but that anti-TIGIT alone did not show statistically significant anti-tumor effects [12, 13]. This may be attributed to differences in the tumor models and differences in the TILs in the TMEs. It is generally accepted that RT promotes the release of chemokines that can recruit immune cells into the TME, including antigen-presenting cells that activate cytotoxic T cells [50]. Conversely, RT can also increase the expression of immunosuppressive receptors or ligands, thereby inhibiting immune responses. Deng et al. found that RT can increase PD-L1 expression in the TME, and that RT combined with anti-PD-L1 therapy can achieve synergistic therapeutic effects, this also establishes a basis for the application of RT combined with immune modulators [26]. Previously, Grapin et al. sought to determine the most effective RT fractionation scheme when combined with anti-PD-L1 and anti-TIGIT therapies; these researchers found no synergistic effect when anti-TIGIT therapy was combined with RT compared to IgG combined with RT [16]. However, in our tumor model, RT combined with anti-TIGIT therapy achieved a good synergistic anti-tumor effect. These differences may be partly due to the different tumor models used, as there were statistically significant differences in the immune microenvironments. For example, anti-TIGIT treatment statistically significant delayed tumor growth in transgenic head and neck squamous cell carcinoma mouse and multiple myeloma models, which enhanced antitumor immune responses by reducing the population of regulatory T- cells (Tregs) [51, 52]. However, Chen et al. and our present study demonstrated that CD4 + T-cells were dispensable [53]. Second, different RT doses have different effects on the number and function of TILs [14, 54]. Combined with existing findings, we conclude that the combination of RT and ICIs still needs to be investigated more comprehensively in order to achieve better efficacy.

DCs are unique immune cells that link innate and adaptive immune responses [55]. Studies have found that elevated TIGIT expression in CD8 + T cells reduces cytokine production and causes poor survival in multiple cancer models. These effects are mediated by DCs through regulation of the TIGIT pathway via the expression of CD155 [4, 12, 56]. These studies provide evidence that the interaction between DCs and TILs may play an essential role in combination therapy. In the present study, we found that DCs expressed a higher level of CD155 in tumor tissues and TdLNs than in normal LNs, and that RT upregulated the expression of CD155 on the surface of DCs; this suggest that CD155 might be a key mediator of DC-mediated T cell suppression in RT. Additionally, we found that combination treatment with RT and anti-TIGIT therapy resulted in a dramatic increase in DCs in the TME. Combining pembrolizumab treatment with toll-like receptor 9 agonists is associated with an elevation in tumor-infiltrating DCs as well as clinical benefits in preliminary studies [57]. Meanwhile, the increased density of conventional DCs (cDCs) in the tumor is associated with improved prognosis in combination with anti-PD1 therapy [58, 59]; this is consistent with our findings. These results indicate that DCs play an important role in the immunotherapy circuits of TIGIT-inhibiting CD8 + T cell responses, and that the development of drugs targeting DCs can contribute to the improvement of anti-tumor efficacy. To our knowledge, this is the first study to demonstrate the key role of DCs in RT combined with anti-TIGIT therapy.

T cell function depends on DC-derived cytokines, including IL-12 and type I interferons [60]. IL-12 is mainly generated by cDCs, with CD8α + and/or CD103 + cDCs as the main subsets [61]. Moreover, tumors grafted onto BATF3^−/−^ mice, which lack cDCs, did not respond to anti-PD-1, anti-PD-L1, or anti-CD137 treatments [36, 62]. Spranger et al. reported that subjects that lack CD103 + DCs within the TME resist the effector phase of the anti-tumor T cell response, contributing to immune escape [46]. In the BATF3^−/−^ mouse model, the anti-tumor effect of RT plus anti-TIGIT combination therapy was absent. A recent study revealed that the activation and accumulation of intratumoral CD8 + effector T cells was dependent on CD103 + DCs in a breast cancer model [35], which was similar to the findings of our study. We also found that the addition of Flt3L significantly increased tumor-specific CD8 + T cell activation at the tumor site and in TdLNs, improving the regression of MC38 tumors. This suggests that Flt3L, RT, and anti-TIGIT triple therapy can promote the induction of tumor-specific CD8 + T cell immunity. Simultaneously, this finding also further explains the indispensable role of DCs in RT plus anti-TIGIT combination therapy, providing a basis for the research and design of new therapeutics.

Published studies have shown that dual ICIs, or ICIs combined with RT, result in a secondary response to tumor re-challenge and reduce the growth of secondary tumors [12, 26]. However, the role of RT combined with anti-TIGIT in this context has not yet been investigated. Our data showed that combination therapy can effectively activate immune effector cells and promote immune memory. In summary, our findings support the hypothesis that blockade of TIGIT combined with RT enhances host anti-tumor immunity in a non-redundant DC-dependent manner and that the presence of DCs may be critical for the function of certain immunotherapies. Nevertheless, the mechanisms underlying the induction of memory responses after the blockade of TIGIT require further investigation.

In summary, our work reveals that RT combined with anti-TIGIT therapy can synergistically exert anti-tumor effects. We found that the combination of RT with anti-TIGIT treatment stimulated CD8 + T cell responses by enhancing local accumulation and modulating cytokine production of DCs via blocking the TIGIT/CD155 axis. The use of Flt3L to increase CD103 + DCs at the tumor site has the potential to improve clinical response to RT and anti-TIGIT treatment in cancer patients. Moreover, our findings explain the mechanism of RT plus anti-TIGIT combination therapy and provide a research basis for the design of new combination therapy strategies. However, in clinical practice, it may not be possible to administer a single dose of 15 Gy to patients. Hence the combined treatment mode of RT dose and anti-TIGIT therapy suitable for clinical application needs to be further explored.

## Supplementary Information

Below is the link to the electronic supplementary material.

Supplementary file1 (DOCX 4346 KB)

## Data Availability

The datasets used and/or analyzed during the current study are available from the corresponding author upon reasonable request.

## Abbreviations

BATF3: Basic leucine zipper transcription factor ATF-like 3
CDCs: Conventional DCs (dendritic cells)
CR: Complete response
CTLA-4: Cytotoxic T lymphocyte-associated antigen-4
DAPI: Diamidinoino-2-phenylindole
DCs: Dendritic cells
ESCC: Esophageal squamous cell carcinoma
Flt3L: Factor FMS-like tyrosine kinase 3 ligand
ICIs: Immune checkpoint inhibitors
Ig: Immunoglobulin
ITIM: Immunoreceptor tyrosine-based inhibitory motif
mAb: Monoclonal antibodies
NCRT: Neoadjuvant chemoradiotherapy
NK: Natural killer
PD-1/PD-L1: Anti-programmed cell death protein 1/programmed cell death ligand 1
RT: Radiotherapy
SEM: Standard error of the mean
TdLNs: Draining lymph nodes
TIGIT: Tcell immunoreceptor with immunoglobulin and ITIM (immunoreceptor tyrosine-based inhibitory motif) domains
TILs: Tumor-infiltrating lymphocytes
TME: Tumor microenvironment
TRG: Tumor regression grade
TVs: Tumor volumes
WT: Wild type
