# Supplementary material for: TIGIT blockade enhances tumor response to radiotherapy via a CD103 + dendritic cell-dependent mechanism
Source: Cancer Immunol Immunother. 2022 Jul 6;72(1):193–209. doi: 10.1007/s00262-022-03227-z (PMC9813151; doi:10.1007/s00262-022-03227-z)

**Supplementary Material**

**Cancer Immunology, Immunotherapy (submitted in 2022) – Zhao et al.**

**Fig. S1** Representative TIGIT staining in human ESCC tissue via multiplex immunohistochemistry in combination with (a) CD8, (b) CD56, and (c) CD4 antibodies.

ESCC, esophageal squamous cell carcinoma; TIGIT, T cell immunoreceptor with immunoglobulin and ITIM (immunoreceptor tyrosine-based inhibitory motif) domains


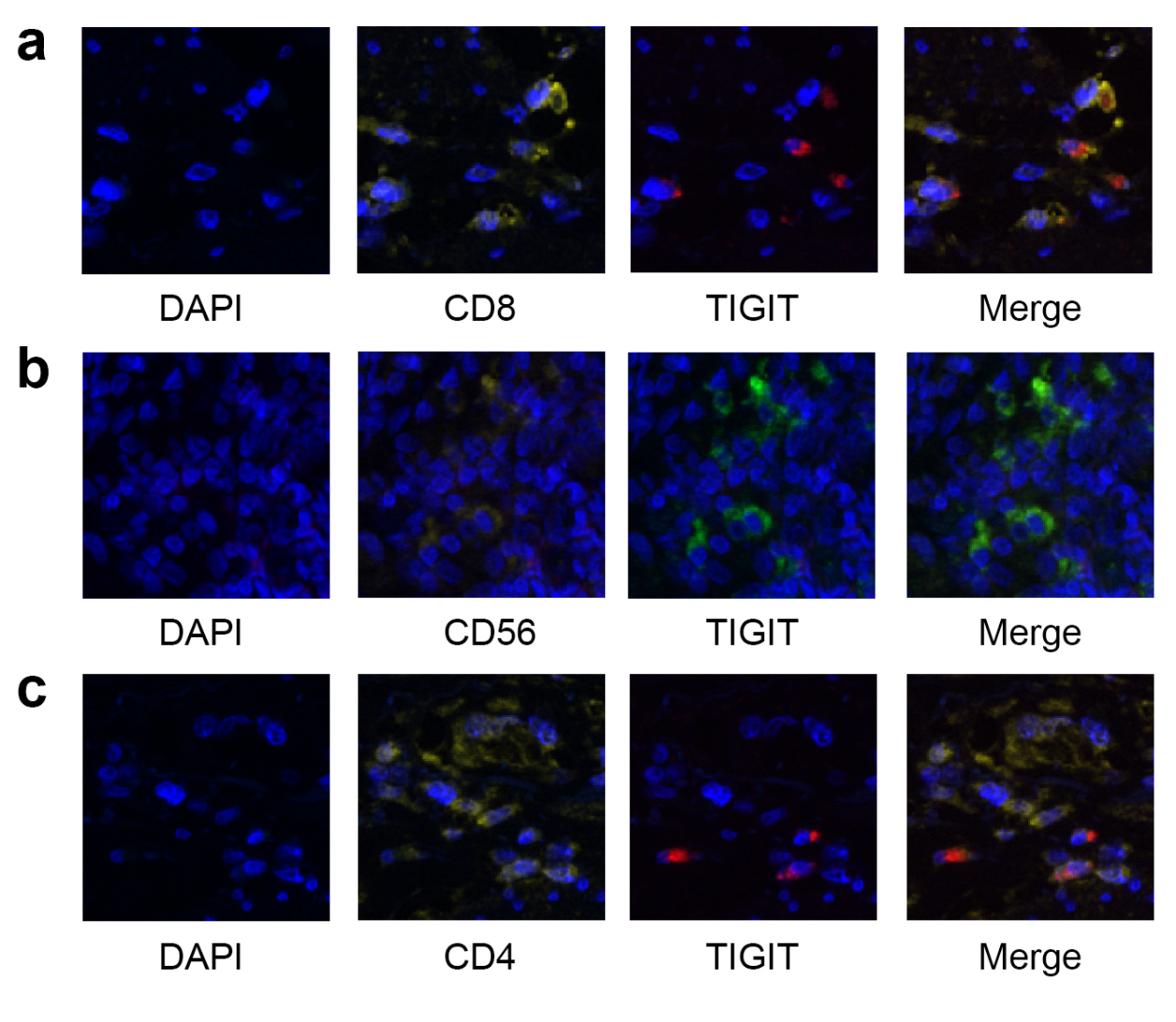


**Fig. S2** TIGIT expression on tumor-infiltrating lymphocytes, TdLNs, and spleen cells in a mouse model. Gating strategy for TIGIT+CD8+T cells, TIGIT+CD4+T cells and TIGIT+NK+ cells (from TILs, TdLNs, and spleen cells) are shown herein.

TdLNs, draining [lymph](javascript:;) [node](javascript:;)s; TIGIT, T cell immunoreceptor with immunoglobulin and ITIM (immunoreceptor tyrosine-based inhibitory motif) domains; TILs, tumor infiltrating lymphocytes


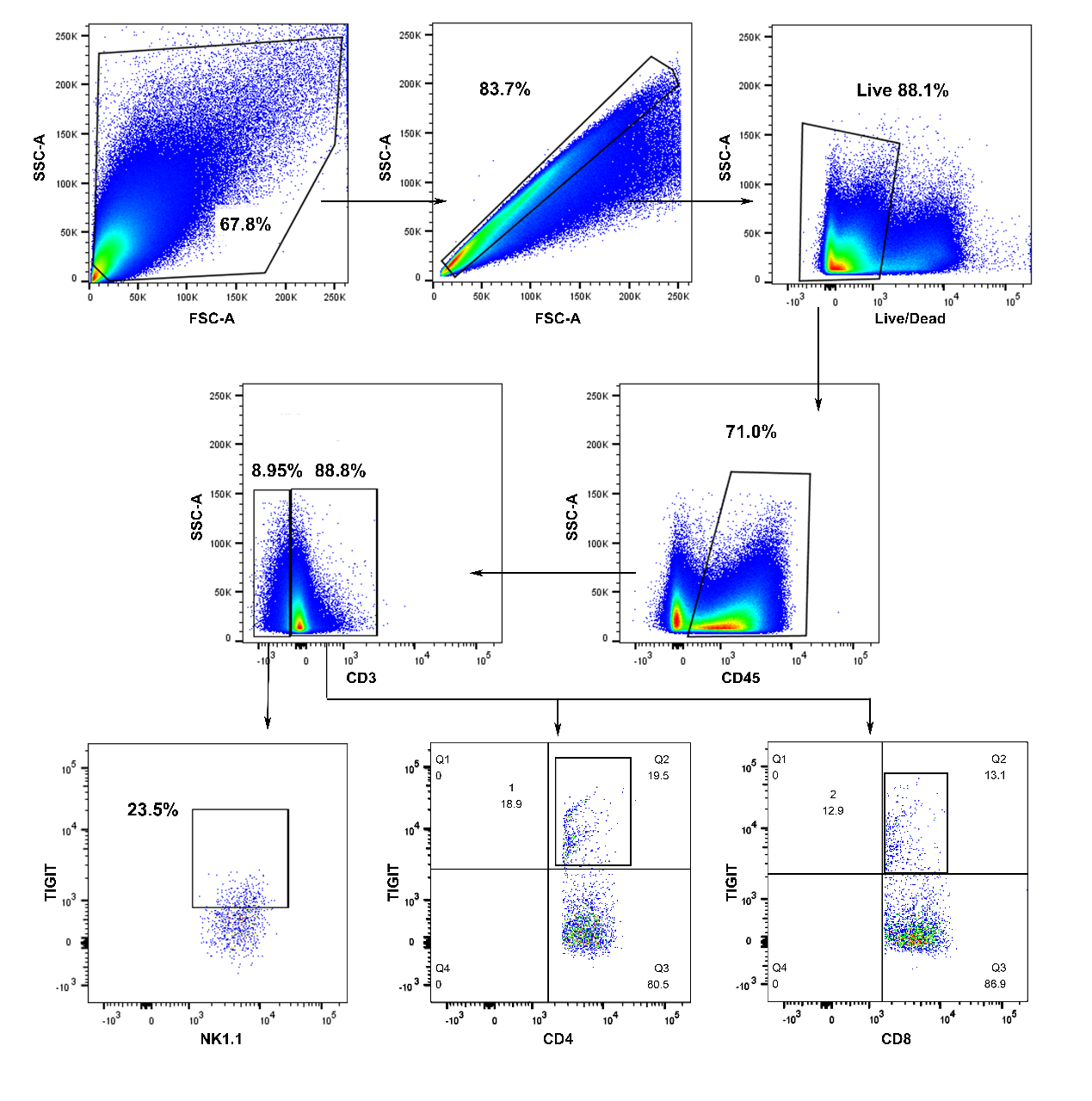


Fig. S3 Change in TIGIT expression on TILs, TdLNs, and spleen cells after RT in mouse LLC and B16 F10 tumor models. **a.** CD8+ T-cells, CD4+ T-cells, and NK cells showed statistically significantly higher expression of TIGIT in LLC TILs and TdLN samples after RT. **b.** CD8+ T-cells, CD4+ T-cells cells, and NK cells also showed increased TIGIT expression after RT in B16 F10 TILs and TdLN samples compared to those that did not receive RT. The spleen samples were unchanged in LLC and B16 F10 tumor models. **p*<0.05; ***p*<0.01;****p*<0.001; *****p*<0.0001

NK, natural killer; NS, not statistically significant’ RT, radiotherapy; TdLNs, draining [lymph](javascript:;) [node](javascript:;)s; TIGIT, T cell immunoreceptor with immunoglobulin and ITIM (immunoreceptor tyrosine-based inhibitory motif) domains; TILs, tumor infiltrating lymphocytes


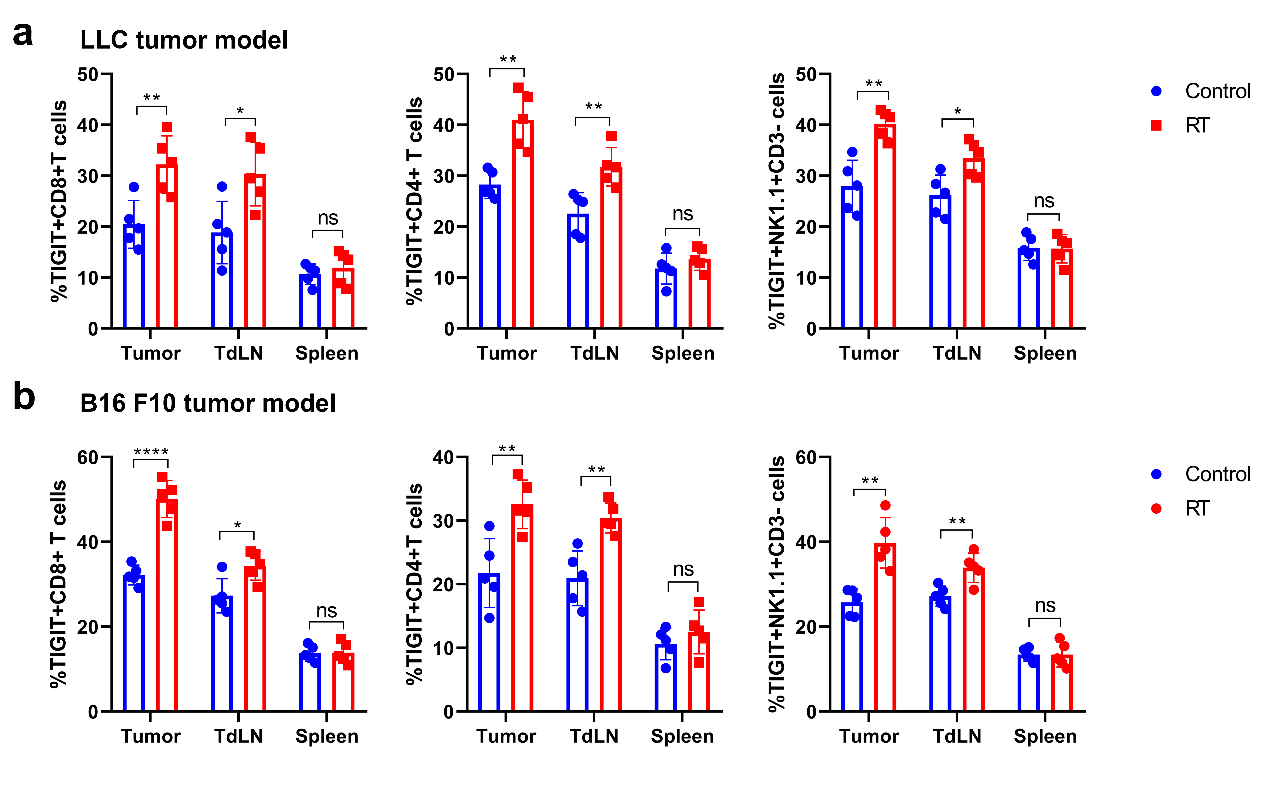


**Fig. S4** RT and TIGIT blockades synergistically elicit tumor rejection. C57BL/6 mice were inoculated subcutaneously with MC38 colon carcinoma cells in their flanks of the abdomen. When tumors reached approximately 70-100 mm^3^ in size, mice were treated with isotype control (blue), anti-TIGIT (red), RT (green), or RT+ anti-TIGIT (purple) antibodies. Individual MC38 tumor volumes are shown over time.

RT, radiotherapy; TIGIT, T cell immunoreceptor with immunoglobulin and ITIM (immunoreceptor tyrosine-based inhibitory motif) domains


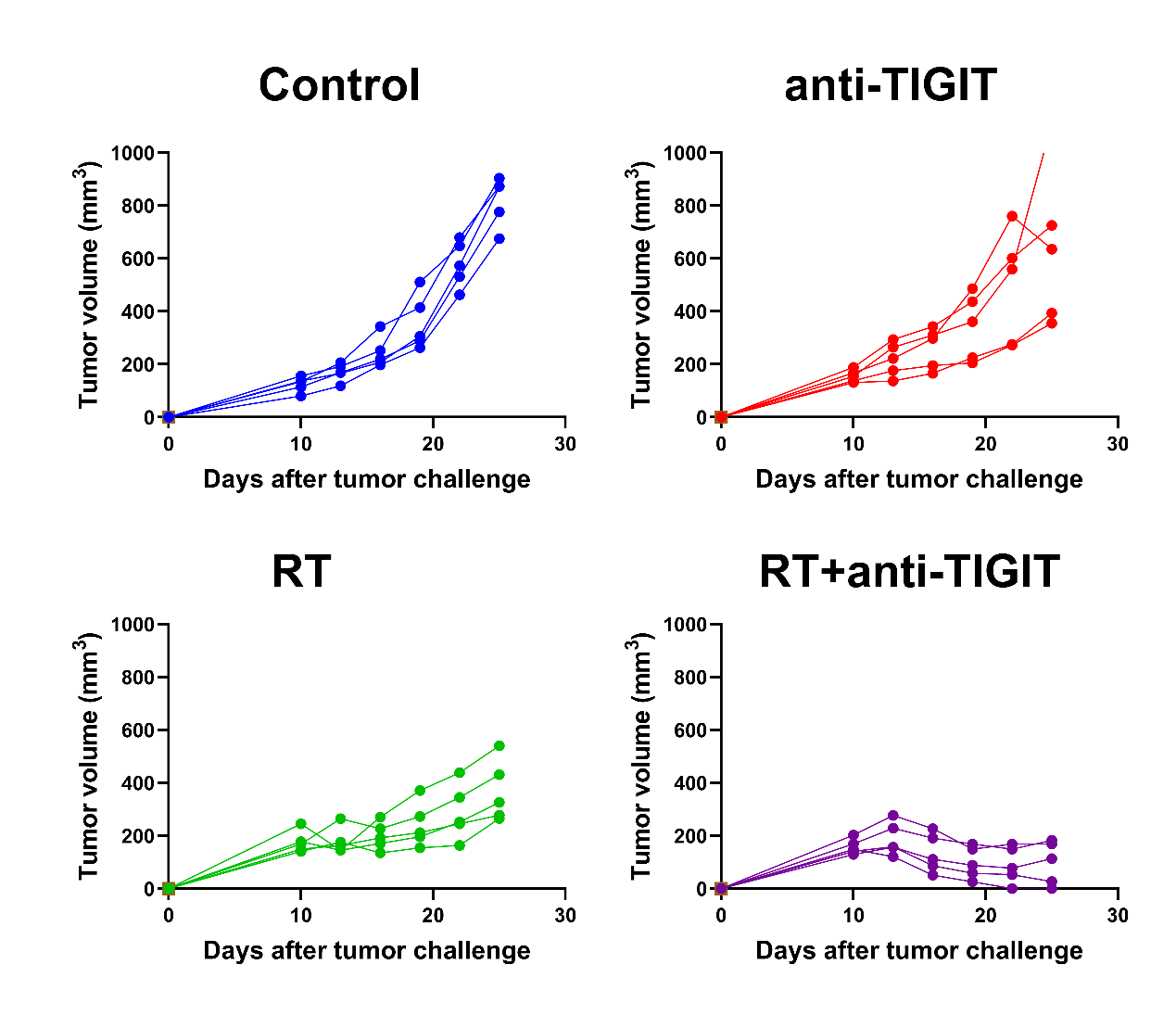


**Fig. S5** Survival curve of the LLC tumor model. The Kaplan Meier survival curve depicts the primary endpoint in mouse models of LLC treated with IgG, anti-TIGIT therapy, RT, and RT combined with anti-TIGIT therapy, respectively. Mouse survival was shown over time. **p*<0.05; ***p*<0.01; ****p*<0.001

IgG, immunoglobulin G; RT, radiotherapy; TIGIT, T cell immunoreceptor with immunoglobulin and ITIM (immunoreceptor tyrosine-based inhibitory motif) domains

**
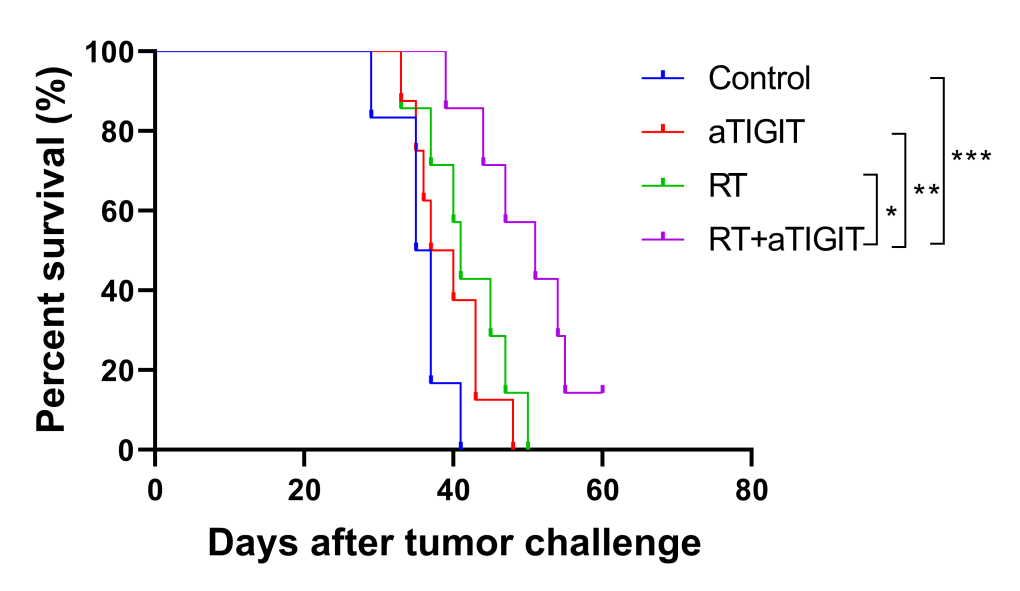
**

**Fig. S6** RT and anti-TIGIT combination therapy improved the observed immune memory effect. Approximately 90 days after the initial inoculation, mice that received RT+ anti-TIGIT therapy and reached CR, as well as naive control mice, were (re)-inoculated with MC38 cells on the opposite flank and inoculated with LLC in their left thoracic flanks. Individual tumor volumes for MC38 and LLC model mice in CR as well as naive mice are shown herein.

CR, complete response; RT, radiotherapy; TIGIT, T cell immunoreceptor with immunoglobulin and ITIM (immunoreceptor tyrosine-based inhibitory motif) domains


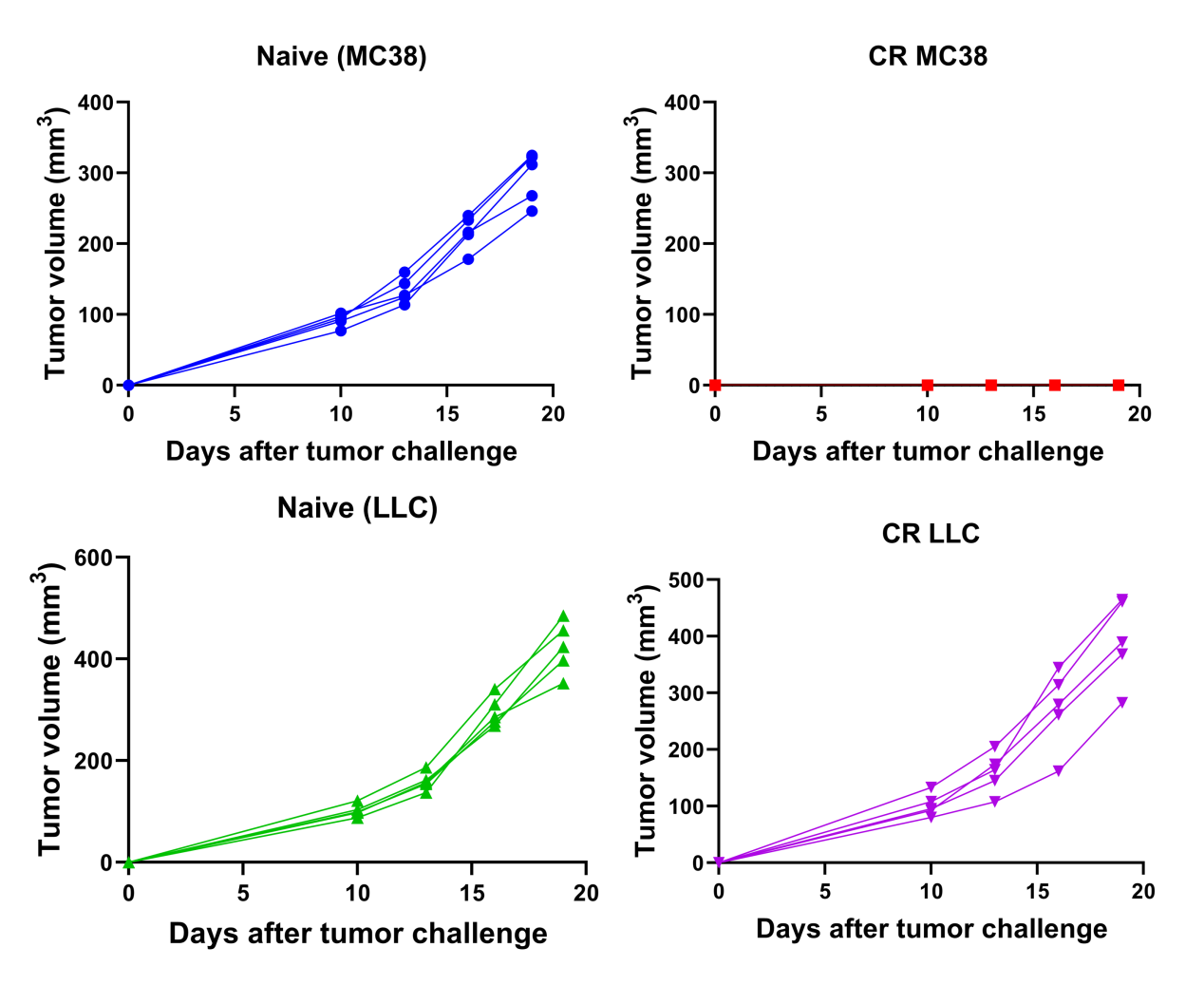


**Fig. S7** CD8+ T-cells are required for effective RT and anti-TIGIT combination treatment. C57BL/6 mice were inoculated subcutaneously with LLC cells in their flanks of the abdomen. When tumors reached approximately 70-100 mm^3^ in size, LLC tumors received 15 Gy and mice were treated with anti-TIGIT therapy (as described in Fig. 3a). Starting from one day prior to RT, 250 μg of depletion antibodies against CD8+ T-cells, CD4+ T-cells, and NK cells were injected intraperitoneally every three days (for a total of four injections). Tumor growth curves are shown herein.*****p*<0.0001

NK, natural killer; RT, radiotherapy; TIGIT, T cell immunoreceptor with immunoglobulin and ITIM (immunoreceptor tyrosine-based inhibitory motif) domains


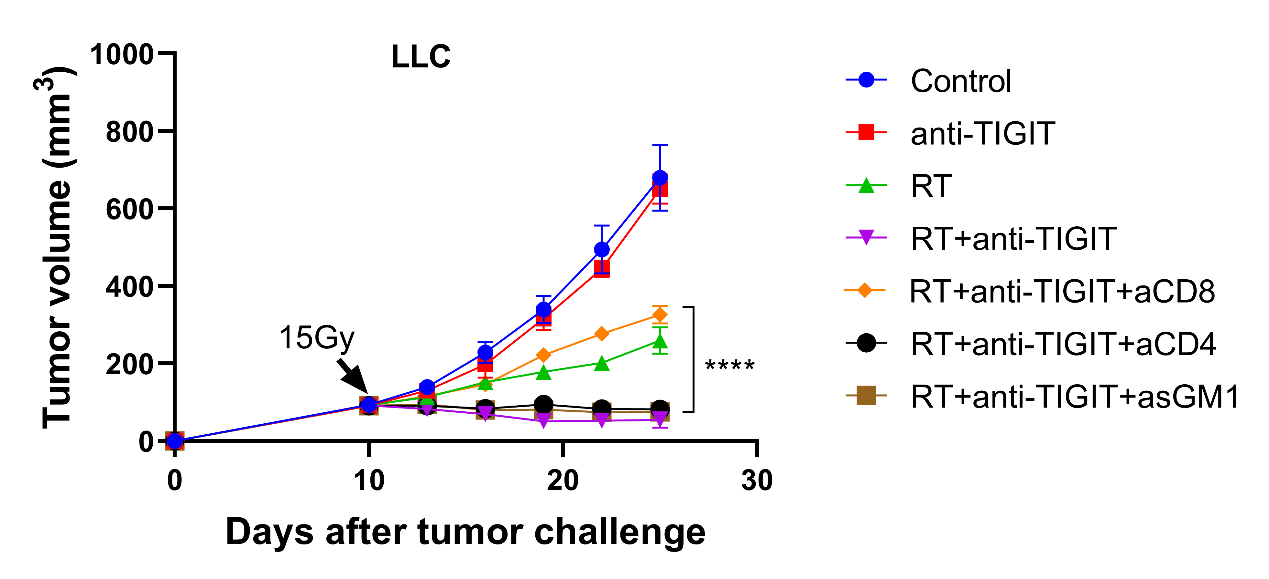


**Fig. S8** Gating strategy: starting cells were gated by FSC/SSC gates. These cells were further gated by single cells and live cells. Next, CD45 leukocytes and CD3+/CD8+, and IFN-γ+/TNF-α+/CD8+ cells were analyzed with regard to the CD45+/CD3+ gate.


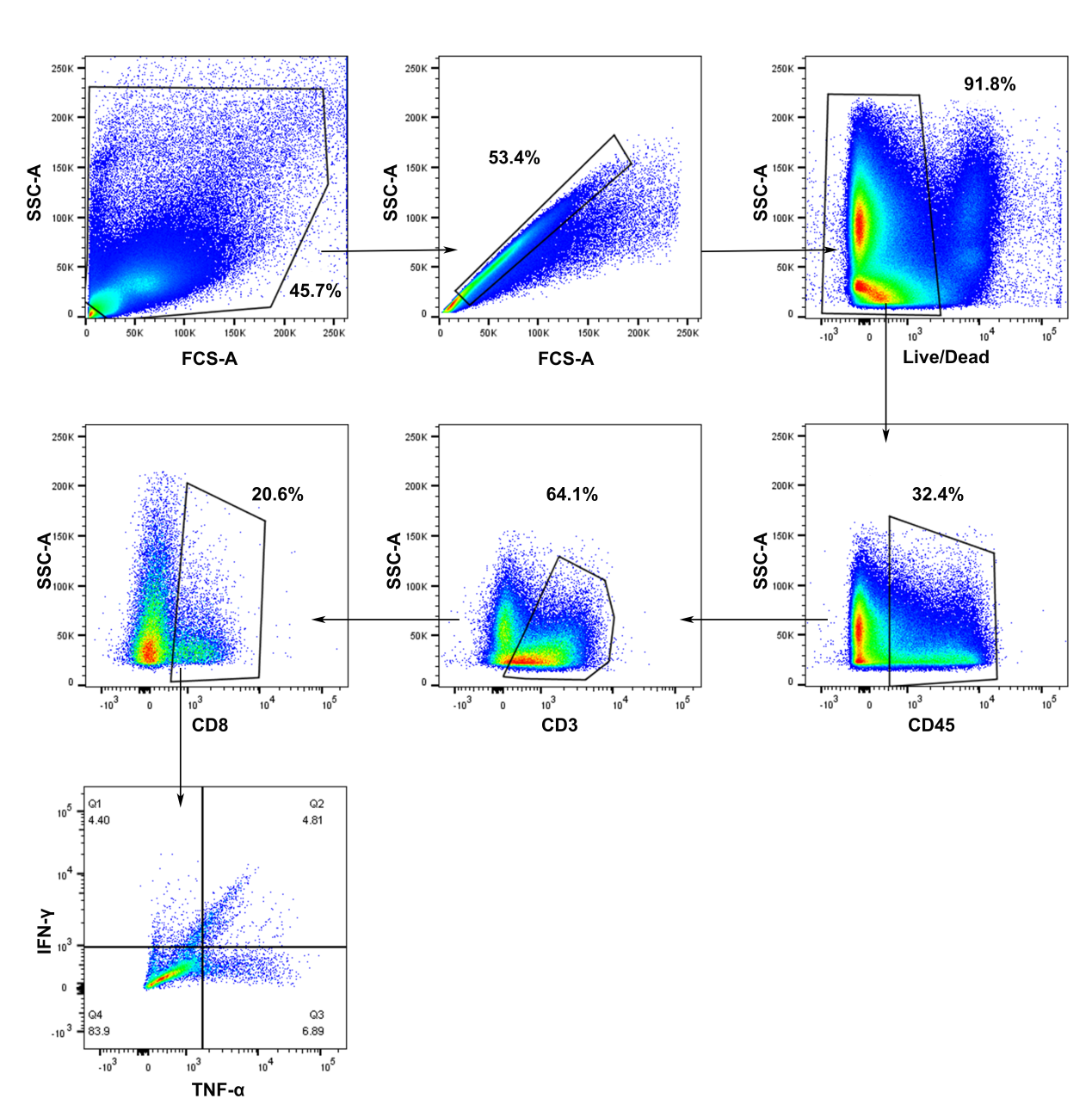


**Fig. S9** Quantitation of IFNγ and TNF-α producing tumor-resident CD8+ T-cells as percentages of total tumor-resident CD8+ T-cells. **p*<0.05; ***p*<0.01; ****p*<0.001; *****p*<0.0001

IFNγ, interferon gamma; NS, not statistically significant; TNF-α, tumor necrosis factor alpha


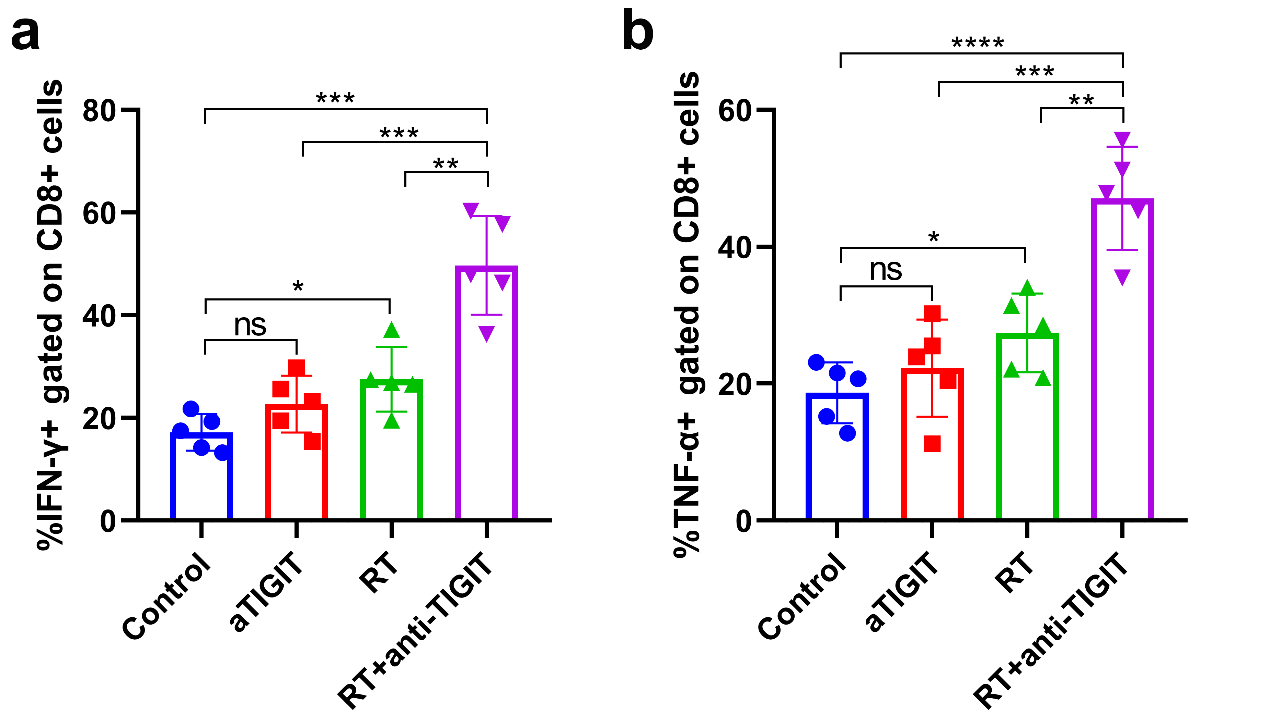


**Fig. S10** Gating strategy: starting cells were gated by FSC/SSC gates. These cells were further gated by single cells and live cells. Next, CD45 (leukocytes) and CD11b-/CD11c+/MHC-II+DCs or CD11c+/MHC-II+/CD103+ DCs were analyzed. IL-10 and IL-12 values were presented in CD11c+ DCs. Histograms of CD155+/CD11c+ DCs and CD155+/CD11c+/CD103+ DCs are shown herein.

DCs, dendritic cells; IL-10, interleukin 10; IL-12, interleukin 12


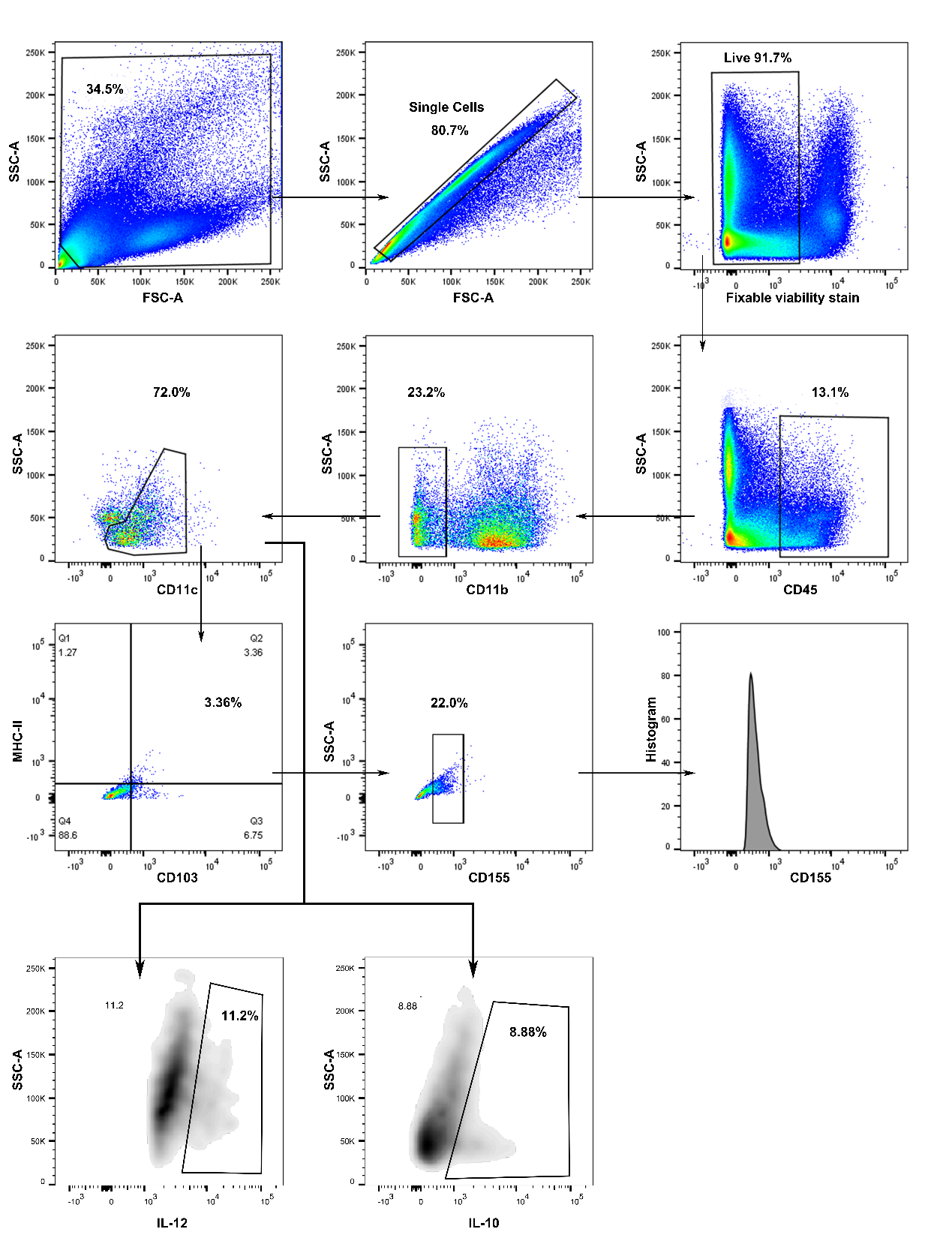


**Fig. 11** The absolute number of CD11c+ DCs standardized to tumor weight. **p*<0.05; ***p*<0.01

DCs, dendritic cells; NS, not statistically significant


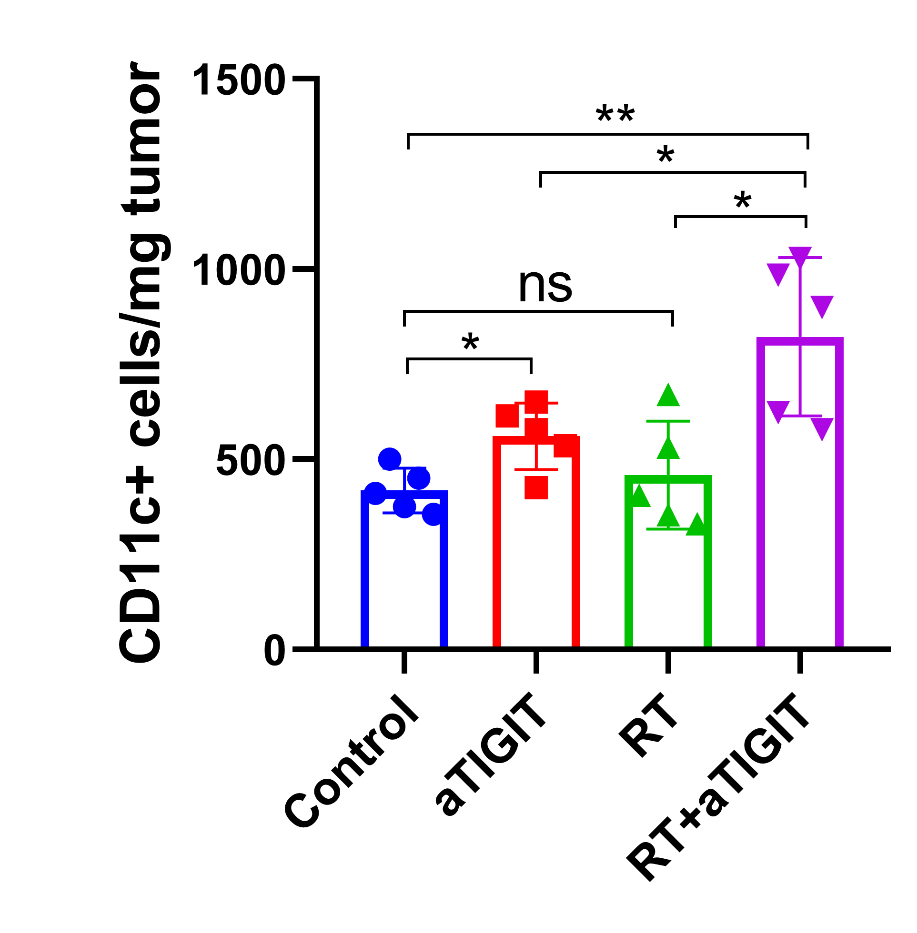


**Fig. 12** FLT3L injections dramatically improved the response of WT mice to treatment with RT and TIGIT mAb. **a,c** Tumor growth is shown for each group. The data are presented as mean tumor growth ± SEM for two independent experiments (n=5). **b,d** Survival times were followed until all experimental mice died. Data shown are from one of two independent experiments performed with similar results. **p*<0.05; ***p*<0.01; ****p*<0.001; ****p*<0.0001

FLT3L, factor FMS-like tyrosine kinase 3 ligand; mAb, monoclonal antibodies; NS, not statistically significant; RT, radiotherapy; SEM, standard error of the mean; TIGIT, T cell immunoreceptor with immunoglobulin and ITIM (immunoreceptor tyrosine-based inhibitory motif) domains; WT, wild-type


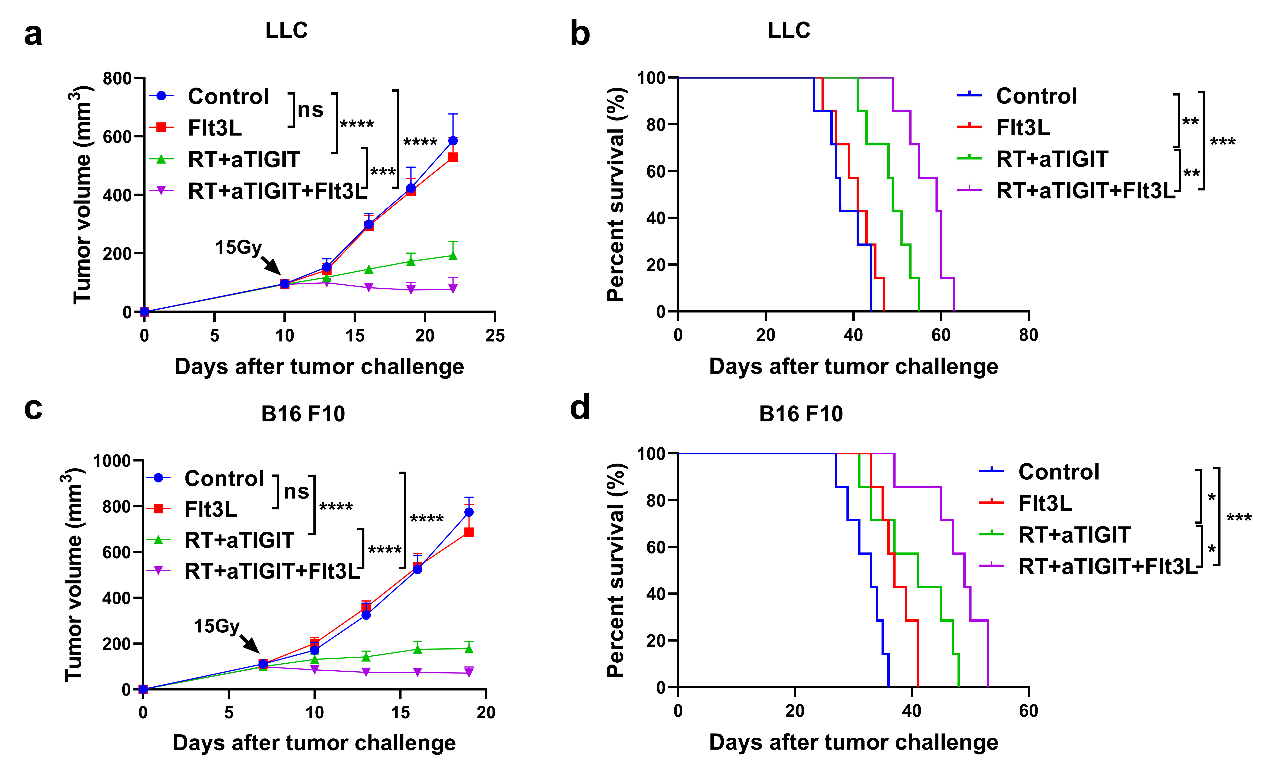

Supplement: Supplementary file 1 — Supplementary file1 (DOCX 4346 KB) [file 262_2022_3227_MOESM1_ESM.docx]
